# Strong succession in arbuscular mycorrhizal fungal communities

**DOI:** 10.1038/s41396-018-0264-0

**Published:** 2018-08-31

**Authors:** Cheng Gao, Liliam Montoya, Ling Xu, Mary Madera, Joy Hollingsworth, Elizabeth Purdom, Robert B. Hutmacher, Jeffery A. Dahlberg, Devin Coleman-Derr, Peggy G. Lemaux, John W. Taylor

**Affiliations:** 1 Department of Plant and Microbial Biology, University of California, Berkeley, CA 94720-3102 USA; 2 Plant Gene Expression Center, US Department of Agriculture-Agricultural Research Service, Albany, CA 94710 USA; 3 University of California Kearney Agricultural Research & Extension Center, Parlier, CA 93648 USA; 4 Department of Statistics, University of California, Berkeley, CA 94720 USA; 5 University of California West Side Research & Extension Center, UC Davis Department of Plant Sciences, Five Points, CA 93624 USA

**Keywords:** Environmental microbiology, Microbial ecology

## Abstract

The ecology of fungi lags behind that of plants and animals because most fungi are microscopic and hidden in their substrates. Here, we address the basic ecological process of fungal succession in nature using the microscopic, arbuscular mycorrhizal fungi (AMF) that form essential mutualisms with 70–90% of plants. We find a signal for temporal change in AMF community similarity that is 40-fold stronger than seen in the most recent studies, likely due to weekly samplings of roots, rhizosphere and soil throughout the 17 weeks from seedling to fruit maturity and the use of the fungal DNA barcode to recognize species in a simple, agricultural environment. We demonstrate the patterns of nestedness and turnover and the microbial equivalents of the processes of immigration and extinction, that is, appearance and disappearance. We also provide the first evidence that AMF species co-exist rather than simply co-occur by demonstrating negative, density-dependent population growth for multiple species. Our study shows the advantages of using fungi to test basic ecological hypotheses (e.g., nestedness v. turnover, immigration v. extinction, and coexistence theory) over periods as short as one season.

## Introduction

Arbuscular mycorrhizal fungi (AMF) are among the most important fungi because they form obligate symbioses that provide phosphorus and nitrogen to 70 to 90% of plant species, including almost all agricultural crops [1, 2]. For more than two decades, the role of AMF as drivers of plant community structure, and vice versa, has been recognized [3–9]. However, owing to the resistance of AMF to cultivation, studies of their ecology have been hampered by controversies over their ability to reproduce sexually, the homogeneity of nuclei in a single individual, and the recognition of AMF species using rDNA regions of different evolutionary rate [10–16].

**Fig. 1:**
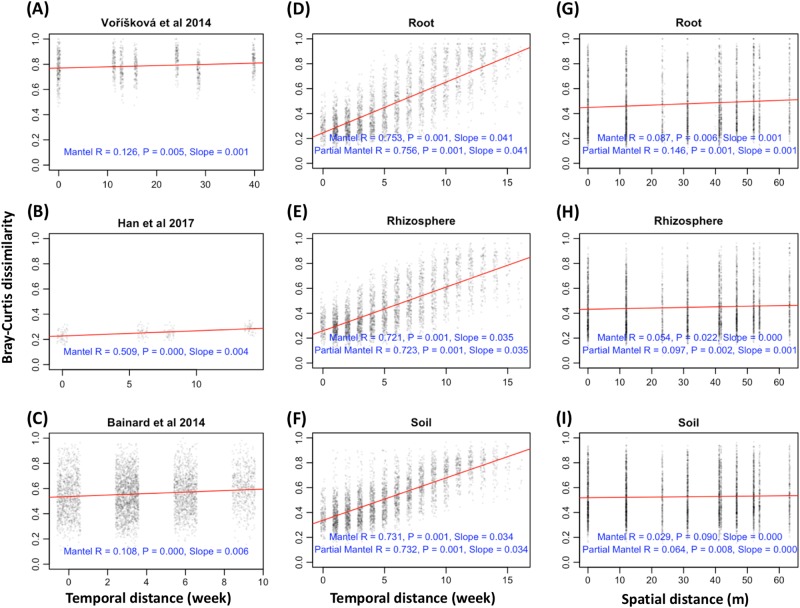
Arbuscular mycorrhizal fungal community change correlated over time (temporal distance **a**–**f**) and space (spatial distance, **g**–**i**). Temporal distance (in weeks between sampling times) as correlated with Bray–Curtis community dissimilarity by Mantel testing in published data from (**a**) [28] [48 samples = 4 time points * 3 vertical layers * 4 plots (10 m^2^ with c. 100 m border)], **b** [29] (21 samples = 3 time points * 7 treatments), and **c** [30] [96 samples = 4 time points * 3 crops * 2 sample type * 4 plots (6 * 2 m^2^ plots with 6 m border)], and from new data presented in this study for (**d**) root (17 time points * 6 plots), **e** rhizosphere (17 time points * 6 plots) and **f** soil (18 time points*6 plots) with all plots having the dimensions 16 m * 8 m with at least a 3 m boarder. Spatial distance as correlated with Bray–Curtis community dissimilarity by Mantel testing from new data present in this study for (**g**) root, **h** rhizosphere, and **i** soil. Note the much stronger association of community dissimilarity and temporal distance reflected by *R* and slope for root, rhizosphere and soil in this study than [28, 29] and [30], and the near absence of association of community dissimilarity and spatial distance in this study. *The [29] result is based on a total fungal community dataset rather than AMF community, due to the low recovery of AMF in that study. Analyses in (**d**–**f**) treat sequence data as counts rarefied among AMF fungi and are nearly identical to analyses treating data as counts rarefied among all fungi or treating data as compositional (Fig. S5)

Detection of succession, the basic ecological process that describes the changes in community similarity over time [17], is one of the ecological investigations most sensitive to species recognition. The changes in communities over time, whether labelled succession or temporal dynamics, have been investigated extensively with modern approaches in plant communities [18–20], studied less extensively in microbial communities [21–26], and are just beginning to be examined with modern tools in AMF [reviewed by Bahram et al [27]; Table S1]. The three most thorough of these studies [reviewed by Bahram et al [27]; Table S1], include two studies that recognized fungal operational taxonomic units (OTUs) with the internal transcribed spacer (ITS) from samples taken either once in each of the four seasons [28], or three times in a single season [29], and a third that recognized AMF OTUs with small subunit (SSU or 18S) ribosomal rDNA from four sampling times from one season [30]. Using data from these three studies to analyze temporal change in fungal community composition, we found a low, albeit significant, rate of change; 0.001–0.006 units of Bray–Curtis dissimilarity per week (Fig. 1a–c).

We hypothesized that the actual rate of change in AMF community composition should be higher than could be detected in these studies dues to two factors. First, temporal change is difficult to detect where variation in the components of the AMF system (plant symbionts, soil, hydration, and season) is large compared to the level of sampling (Table S1). Second, change in community composition is under reported when AMF species-level OTU recognition relies on the conserved SSU ribosomal rDNA [2, 10, 31, 32], rather than on the more variable ITS, which is the molecular “barcode” region used for OTU recognition in almost all other fungal studies [33–36].

Here we revisit the basic ecological process of succession by (i) using a system with low environmental heterogeneity comprising only one soil type, one irrigation scheme, two cultivars of the agricultural host plant, sorghum [*Sorghum bicolor* (L.) Moench], and weekly, triplicate sampling of soil, rhizosphere, and roots throughout the 17 weeks from seedling emergence through grain maturation, and (ii) using OTUs characterized with ITS2 by a recently published approach [37, 38] (Database S1-S2). Our data show a signal of succession in AMF communities that is more than an order of magnitude larger than previously reported. To understand the basis for this signal, we explore patterns of the nestedness and turnover, the processes of appearance and disappearance (proxies for the processes of immigration and extinction that are appropriate for microbial, HTS datasets), and ask if the processes are deterministic or stochastic, and positively or negatively dependent on initial population size.

## Methods

### Sampling and sequencing

This experiment was conducted at the semiarid Kearney Agricultural Research and Extension (KARE) Center in Parlier, CA, USA (36.6008° N, 119.5109° W). Two sorghum [*Sorghum bicolor* (L.) Moench] cultivars with similar flowering times, RTx430 and BTx642, were planted in three, separate, 16×8 m^2^ plots (each with ten rows) with 3 m borders between plots (Fig. S1), and were watered using drip irrigation with 80% of calculated evapotranspiration on a weekly basis [39]. The trial was planted on 27th May 2016 and plants emergence was recorded on 1st June. Weekly samples of root, rhizosphere and soil were taken in 2016 on June 8, 15, 22, 29; July 6, 13, 20, 27; August 3, 10, 17, 24, 31; and September 7, 14, 21, 28. At each sampling time, ten or more individual sorghum plants were removed from randomly chosen locations within one of the central eight rows in each plot and combined to generate one root sample and one rhizosphere sample. At the same time, ten soil cores were taken from random locations in each plot and combined to generate one soil sample. Thus, a total of 312 samples were taken, which comprise 17 weekly samples of the two cultivars, and three compartments (root, rhizosphere, and soil), all with three replicates, plus six soil samples collected prior to planting. DNAs of root, rhizosphere, and soil samples were extracted using the MoBio PowerSoil DNA kit (MoBio, Carlsbad, CA, USA). The fungal internal transcribed spacer 2 (ITS2) region was amplified using forward and reverse primers designed to contain a 29 (forward) or 25 (reverse) base linker, a 12 base barcode, a 29 (forward) or 34 (reverse) base pad, a 0–8 base heterogeneity spacer [40], and either the fungal ITS2 specific 21 base 5.8SFun primer (forward) or 27 base ITS4Fun primer (reverse) [38] (Table S2). We used Lee Taylor’s ITS2 primers [38] because the 5.8SFun and ITS4Fun matched well with all Glomeromycotina lineages when we matched the primers with published SSU-ITS-LSU alignments [41] (Database S1–S2). All the raw sequences are deposited in Sequence Read Archive with the accession codes: Bioproject PRJNA412410 Biosamples SAMN07711256 - SAMN07711567. Detailed information about site description, experiment design, and molecular analysis can be found in the supplementary methods.

### Bioinformatics

Overall sequencing quality was evaluated using FastQC v0.11.5 [42]. Forward and reverse reads were merged using the fastq_mergepairs command (-fastq_maxdiffpct 3) in USEARCH v8.0 [43]. Primers were removed using cutadapt v1.9.1 [44]. Quality control was carried out using the fastq_filter command (-fastq_maxee 1.0 -fastq_minlen 200) in USEARCH [43]. High quality sequences were subjected to de-replication and de-singleton, and then clustered into OTUs using the cluster_otus command in USEARCH [43]. The OTUs were searched against the raw reads using the usearch_global command (-id 0.97) in USEARCH [43]. This step generated a table of 312 samples×1293 OTUs (10,770,762 reads). The representative sequence of each OTU was identified by a BLAST search against the curated, fungal specific UNITE database [45] and the NCBI database. Fifty-two OTUs (167,749 reads) were identified as AMF (Table S3), whereas 1026 OTUs were non-AMF (10,341,780 reads) and 215 were non-fungal (261,233 reads). To use phylogenetics to equate AMF OTUs with known species, sequences representing the 52 AMF OTUs were combined with vouchered sequences downloaded from NCBI and UNITE and all sequences were aligned using MAFFT v 7.310 [46] placed in a neighbor-joining tree using MEGA v8.0 [47]. Representative sequences of AMF OTUs were deposited in GenBank with the accession codes: MG008508 - MG008559. Owing to the possibility of multiple ITS2 sequences within an individual AMF [16], we searched for OTUs with identical read abundance by analysis of variance (ANOVA). For OTUs with no difference in abundance, a series of pairwise correlations was then carried out  and those OTUs with equal abundance and strong positive correlation were combined to avoid the issue of multiplicity of ITS2 sequences within individual AMF.

### Statistical methods

Recent recognition that microbiome data from high-throughput sequencing (HTS) represents a random sample of the DNA molecules in an environment and not absolute counts of the molecules dictates that the data be treated as compositional [48] and not as counts, as commonly has been done. Therefore, we use one compositional and two traditional approaches to analyze our AMF data to both analyze the data as compositional and to permit comparisons with prior studies. For the first traditional approach (dataset 1), we rarefied the number of AMF sequences per sample to 100 using the rrarefy command in vegan in R [49, 50], an approach designed to eliminate the effects of different read numbers among the samples on the deduced AMF community composition. For the second traditional approach (dataset 2), we rarefied all fungal reads to 2743 and then extracted the AMF subset of this normalized fungal data, an approach designed to eliminate the effects of different fungal read numbers but retain the abundance variation of AMFs among the samples. For the compositional method (dataset 3), we imputed zeros in AMF compositional count data sets based on a Bayesian-multiplicative replacement using the cmultRepl command in zCompositions [51], and then converted these data to the centered log-ratio (CLR) using the codaSeq.clr command in CoDaSeq (https://github.com/ggloor/CoDaSeq) [48]. We present analyses of the three datasets in figure and supplemental figures to invite comparison. Direct comparison is possible with permutation tests for ANOVA (PERM ANOVA), but not for other analyses because the statistical methods for compositional datasets are different from those for traditional count datasets, e.g., Bray–Curtis dissimilarity for counts v. Aitchison distance for compositional, and principal coordinate (PCo) analysis for counts v. principal component (PC) analysis for compositional [48]. For some of our analyses, methods are not yet available for compositional datasets, e.g., partition of nestedness and turnover components of beta diversity [52].

By plotting time and AMF richness (dataset 1), we demonstrated the temporal dynamics of AMF diversity. To assess the phylogenetic relatedness of AMF OTUs within every sample, the net relatedness index  (NRI) was calculated based on the above-mentioned phylogenetic trees and community composition data using the ses.mpd command (×−1) in picante package [53]. Relationships between time and abundance of initially dominant and initially rare OTUs (dataset 1, 2, 3) were explored by linear mixed-effects models, including random effects of OTU identity using the lme command in the lme4 package [54]. The variance explained (conditional *R*^*2*^) by the mixed effect models was calculated by the r.squaredGLMM function in MuMIn Package [55].

Bray–Curtis dissimilarities were calculated for dataset 1 and 2 to construct distance matrices of the AMF community (Hellinger transformed) using the vegdist command in vegan [49], and Aitchison distances were calculated for dataset 3 [48]. PERM ANOVA were carried out to assess the effect of compartment (soil, rhizosphere or root), time period and cultivar on the AMF community variation either detected by Bray–Curtis dissimilarities or Aitchison distances using the adonis command in vegan [49]. Euclidean dissimilarities were calculated to construct distance matrices of geographic, temporal, temperature, and solar radiation distances respectively in vegan [49]. Mantel tests were carried out to explore the correlations between these distance matrices [49]. Partial Mantel tests were carried out to explore the relationships between AMF community dissimilarity and temporal distance, after excluding the influence of geographic distance. Conversely, partial Mantel tests were carried out to explore the relationships between AMF community dissimilarity and geographic distance, after excluding the influence of temporal distance. Structural equation models (SEM) using Mantel *R* values as input were constructed in AMOS 25.0 [56] to explore the causal relationships among time, solar radiation, temperature, plant biomass and AMF community composition. Based on a priori and theoretical knowledge, we assumed a conceptual model in which time and solar radiation affect temperature, which in turn affects plant biomass, which further influences AMF community composition. To test the homogeneity of AMF community during succession [57], beta dispersion of AMF communities was explored by the betadisper function in vegan [49]. To graphically illustrate the AMF community composition, AMF Bray–Curtis dissimilarity matrices were ordinated by PCo analysis using the pcoa command in the Ape package [58], and AMF Aitchison distance were ordinated by PC analysis using the prcomp command in stats package [50]. The turnover and nestedness components of AMF community were calculated based on the presence/absence data using the beta.pair command (index.family = ‘sorensen’) in the betapart package [59], and were fitted with temporal distance using the Mantel test in vegan [49]. The nestedness of AMF community was graphically illustrated by the nestedtemp command in vegan package [49].

To test how the AMF succession might be influenced by the AMF OTU cutoff, the OTU delineation processes were repeated by changing the OTU cutoff from the defaulted 97 to 80% in increments of 1%. We calculated the AMF community Bray-Curits dissimilarity of every OTU cutoff, and fitted it with temporal distance using Mantel test, as mentioned above.

To compare the temporal dynamics of AMF communities in our study with those previously reported by Bainard et al. [30], Han et al. [29] and Voříšková et al. [28], we calculated, for the three previous studies, Bray–Curtis dissimilarities of Hellinger transformed AMF community data, and Euclidean dissimilarities of temporal distance in terms of simulated weekly sampling. Mantel tests were carried out to explore the correlations between AMF community dissimilarity and the temporal distances in vegan [49].

## Results and discussion

### Recognition of AMF OTUs by ITS2

To recognize AMF OTUs that approximate species more closely than SSU OTUs we use the ITS2 region of the RNA repeat [10, 32, 37, 60]. Here, using Illumina Miseq of fungal ITS2 amplified by dual-barcoded Lee Taylor’s fungal specific primers [38], we successfully recognized 52 AMF OTUs with 167,749 AMF reads, belonging to *Glomus* (21 OTUs), *Rhizophagus* (13 OTUs), *Claroideoglomus* (8 OTUs), *Funneliformis* (5 OTUs), *Paraglomus* (4 OTUs), and unidentified Glomeraceae (1 OTUs) (Fig. S2). The thorough sampling (312 samples) produced a species accumulating curve that reached its plateau for the species-poor AMFs (52 OTUs) in a relative small (<5000 m^2^), simple agricultural field (Fig. S3). In line with this result, of the 52 AMF OTUs, only five occurred in fewer than 10 samples, suggesting a lack of rare OTUs in our study (Fig. S4).

As described in the section on statistical methods, to both recognize the compositional nature of HTS microbiome data [48] and to permit comparisons of our results with previous studies that treat HTS data as counts, we analyzed the data both as counts and compositional. We employed two count methods: in data set 1 we rarefied to equal AMF reads and in dataset 2 we rarefied to equal fungal reads. For compositional analysis, in dataset 3 we transformed the data by the CLR method [48]. The largest difference is the detected effect of time, *R*^*2*^ = 0.438 for dataset 1, *R*^*2*^ = 0.339 for dataset 2 and *R*^*2*^ = 0.232 for dataset 3 as explored by PERM ANOVA (all *P* < 0.001) (Fig. S6). Despite these differences, analyses of the three different datasets generated remarkably consistent results in all applicable analyses that similarly supported the main conclusions of our study (Fig. S5–S7).

In light of recent reports of ITS2 variation as high as 6 to 12% in AMF species-level clades [16, 61, 62], we also investigated the effect on ecological analyses of reducing, in 1% increments, the threshold of OTU recognition by ITS2. We found that the rate of AMF succession was not substantially changed until the cutoff was reduced from 97 to 85% (15% intra-OTU variation, Fig. S8), therefore, our findings are not affected by the potential intraspecific variation reported for AMF species.

The use of ITS has been questioned due to reports showing that one AMF individual can contain more than one, independently evolving rDNA repeat [10, 16, 32]. Mindful of the possibility of amplifying and sequencing more than one rDNA repeat in a single species of Glomeromycotina, we searched for possible intra-individual rDNA polymorphism by correlating read abundance for the different ITS2-OTUs over the 17 weeks of sampling. Strongly correlated ITS2 read abundance (Fig. S9) was seen for three *Rhizophagus* OTUs (118, 161, 132). Therefore, due to the possibility that they might represent a single AMF species, we treated these three *Rhizophagus* OTUs as a single species in our analysis, reducing the number of ITS2-OTUs from 52 to 50. Two other *Rhizophagus* OTUs showed similar read-abundance patterns (Fig. S9A) but the unequal abundance of reads (Fig. S9B) indicated that they represented distinct OTUs and we retained them in our analyses. To assess the effect of reducing the number of OTUs from 52 to 50, we repeated the following ecological analyses with all 52 ITS2-OTUs, finding no differences in ecological results, their significance or our subsequent conclusions (Figs. S10–S15).

### Succession of AMF community

**Fig. 2:**
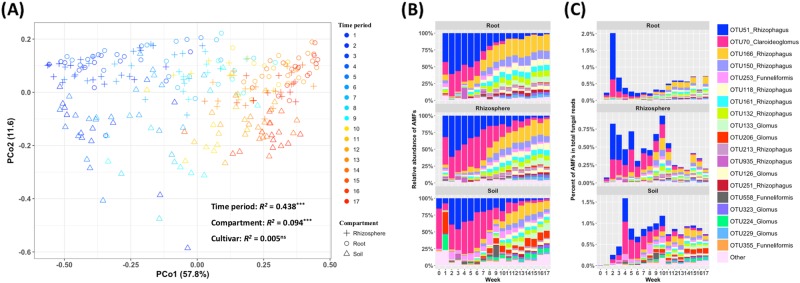
Change in composition of arbuscular mycorrhizal fungal communities in three compartments (root, rhizosphere, and soil) over 17 weekly time period (TP) samplings. **a** Principal coordinate (PCo) analysis by PERM ANOVA showing significant association of community composition with time period (TP) and compartment but not cultivar (***P < 0.001; ns: not significant). Note that TP accounts for nearly half the variance, which is far more than is accounted for by compartment (root, rhizosphere or soil) or plant genotype (sorghum cultivar RTx430 or BTx642). **b** Bar graph of AMF operational taxonomic unit (OTU) relative abundance at each TP and **c** Bar graph of AMF OTUs percentage in total fungal reads at each TP for the three compartments, root, rhizosphere and soil. Note the strong change in AMF community composition over time. Analysis in  **a** treats sequence data as counts rarefied among AMF fungi and is nearly identical to analyses treating data as counts rarefied among all fungi or treating data as compositional (Fig. S6)

Our analyses showed a strong, positive, Mantel correlation (*R* = 0.617–0.753, *P* < 0.001) between temporal distance (graphed on the *x*-axis as weeks between sampling times) and AMF community Bray–Curtis (dataset 1, 2) or Aitchison (dataset 3) dissimilarity in root, rhizosphere, and soil samples (Fig. 1d–f; Fig. S5). The slope of the change in dissimilarity per week found here (0.034–0.041 units of Bray–Curtis dissimilarity per week, dataset 1), is 34–41 times greater than the first previously mentioned study (0.001 units of Bray–Curtis dissimilarity per week, Fig. 1a) [28], 8.5–10.25 times greater than the second (0.004 units of Bray–Curtis dissimilarity per week, Fig. 1b) [29], and 5.6–6.8 times greater than the third (0.006 units of Bray–Curtis dissimilarity per week, Fig. 1c) [30]. This change in AMF community composition can also be visualized by ordination (PCo for dataset 1, 2; PC for dataset 3) analysis (Fig. 2a; Fig. S6), by a proportional bar plot of AMF relative abundance (dataset 1, Fig. 2b), or by a bar plot of percentage of AMFs in total fungal reads (dataset 2, Fig. 2c), in addition to the graph of community dissimilarity and temporal distance (Fig. 1d–f; Fig. S5; Fig. S8; Fig. S10). This strong AMF succession was also seen using PERM ANOVA (*R*^*2*^ = 0.232–0.438; *P* < 0.001, Fig. 2a; Fig. S6). This succession is not confounded by beta dispersion in root, rhizosphere, and soil (Fig. S16). We recognize that the concept of succession, which was developed for plant communities, is controversial when applied to microbial communities. Here, we adopt a recent definition of succession as, “… somewhat orderly and predictable manner by which communities change over time following the colonization of a new environment…” [22], by treating a newly emerged plant root, as well its associated rhizosphere and soil, as new environments for AMFs to colonize and initiate succession.

Geographic distance is a factor known to have a major effect on AMF community composition [12, 27, 63]. In contrast to temporal distance, our analysis of the effect of geographic distance using Mantel and partial Mantel tests showed a small effect (slope of the change in dissimilarity over distance = 0 to 0.001 per meter, *R* never greater than 0.15) on the variation of AMF community dissimilarity in root, rhizosphere, and soil (Fig. 1g–i). Thus, we can infer that agricultural cultivation of a single plant species (*S. bicolor*) homogenizes AMF communities over at a range of from 10 to 60 m, but we cannot rule out environmental heterogeneity that might occur at finer scales and that could affect AMF community composition.

**Fig. 3:**
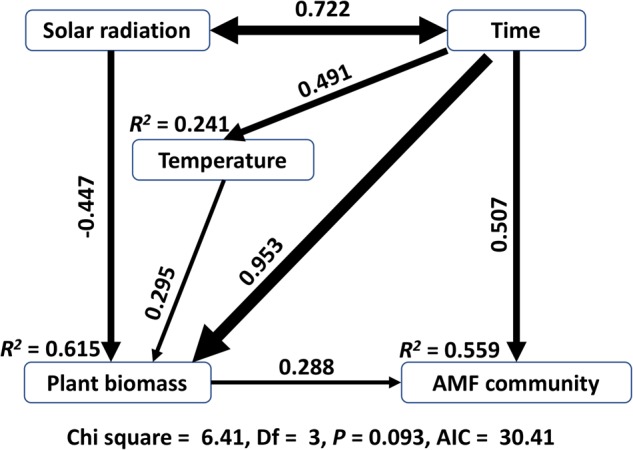
Structural equation model (SEM) demonstrates that the succession of arbuscular mycorrhizal fungal (AMF) communities was directly affected by time and aboveground biomass of sorghum, in addition to indirect (via plant biomass) effects of solar radiation and temperature. The numbers above the arrows indicate the magnitude of path coefficients (*λ*), and this magnitude is also depicted by the width of the lines. *R*^*2*^ values represent the proportion of variance explained for each variable

AMF community ecology follows approaches developed for plants with a major difference being the immediate source of energy, insolation for plants and symbiotic partners for AMF [1], sorghum in our case. Of course, temporal variation in insolation that directly affects the plant symbiont should have an indirect effect on AMF. Our SEM results showed that AMF community was directly affected by time and plant biomass, and also indirectly by temperature and solar radiation (Fig. 3). Surprisingly, solar radiation negatively affected plant biomass. It might be that at 36˚N latitude in Central California, energy from insolation is not a limiting resource for sorghum growth, but UV radiation and drought stress associated with high insolation might detrimentally affect accumulation of sorghum biomass.

### Nestedness and turnover during AMF community succession

**Fig. 4:**
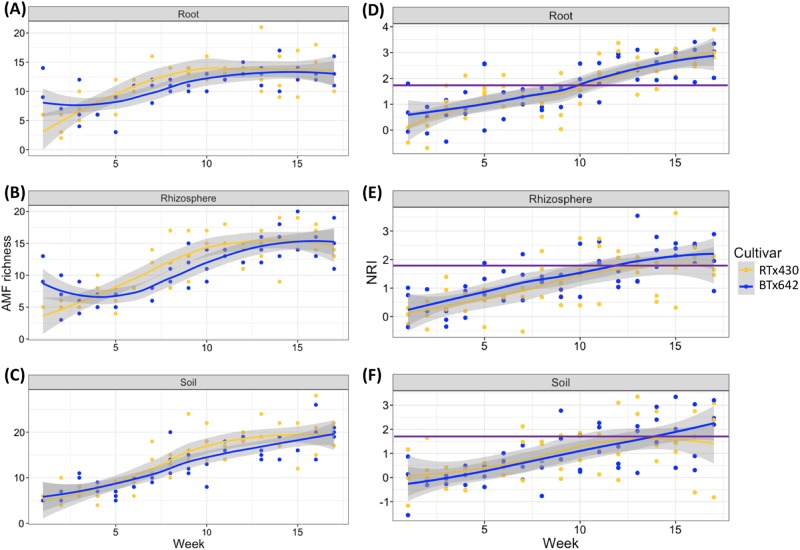
Temporal dynamics of (**a**–**c**) richness and (**d**–**f**) phylogenetic relatedness of AMF communities on two sorghum cultivars. Richness shows a consistent increase over time for all three compartments (root, rhizosphere, and soil). Phylogenetic relatedness (net relatedness index, NRI) also increases over time, eventually showing significant underdispersion as it rises above the threshold of significance (horizontal, purple line). Note that the threshold is reached earlier inside roots than outside them in the rhizosphere and soil and that both cultivars (RTx430 and BTx642) behave similarly in terms of richness and NRI, consistent with the analyses in Fig. 2a

**Fig. 5:**
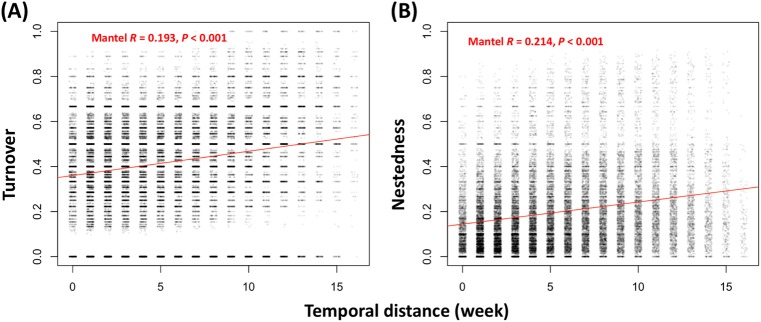
Role of two patterns, (**a**) turnover and (**b**) nestedness in the change in AMF community composition over time. The compositional variance of AMF community measured by Sorenson pair-wise dissimilarity was partitioned into a turnover component (Simpson pair-wise dissimilarity) and a nestedness component (Sorenson pair-wise dissimilarity minus Simpson pair-wise dissimilarity) following Baselga [52]. Subsequently, Mantel tests were carried out to explore the correlation of temporal distance and either the turnover or nestedness components of AMF compositional variance. Both AMF turnover and nestedness showed significant and biologically meaningful associations with temporal distance. Visualization of the superimposed points was enhanced by rendering them semi-transparent and adding a small amount of noise to the temporal distances

There are two, divergent patterns describing the change in community composition: turnover (where some species are replaced by others over time) and nestedness (where the earlier community is a subset of the latter community, or vice versa) [52]. Our demonstration of AMF community succession (Figs. 1d–f, 2) was accompanied by an increase in richness (Fig. 4a–c) over the 17 weeks from emergence of seedlings to maturation of grain in sorghum, so we expected nestedness to predominate but questioned if replacement (turnover) also was involved. Mantel tests showed that temporal distance was significantly correlated with both the components of turnover (*R* = 0.193, *P* < 0.001) and nestedness (*R* = 0.214, *P* < 0.001) of AMF community composition variation (Fig. 5; Figs. S17–S19). The co-occurrence of these two divergent patterns of change in community composition suggested that there also would be more than a single, ecological process underlying succession in the AMF community.

### Immigration and extinction in AMF community succession

Immigration and extinction are the two fundamental processes responsible for the patterns of succession [18]. Although immigration and extinction are far more easily observed for plants than microscopic fungi, our comparison of the first week (Time Period 1, TP01) and the last week (Time Period 17, TP17) provide evidence for both processes. Two initially dominant TP01 OTUs with indicator values (*indval*) strong enough to make them significant indicators of the initial time period (OTU51_*Rhizophagus*, *indval* = 0.894, *P* < 0.001; OTU70_*Claroideoglomus*, *indval* = 0.809, *P* < 0.001) were subsequently lost and 13 initially rare OTUs (five *Rhizophagus*, *indval* = 0.667–0.811, *P* < 0.001; eight *Glomus*, *indval* = 0.311–0.816, *P* < 0.05) became significant indicators by the final sampling at TP17 (Table S4; Fig. 2b). This result was seen with abundance of AMF alone or abundance of AMF relative to all fungal, although in the later analysis, the initial dominance was delayed from TP01 to the 2nd week in root and rhizosphere and the 4th week in soil (Fig. 2c).

Again acknowledging the difficulty of asserting the absence of a microscopic fungus, the loss of the initially dominant OTUs is consistent with the action of forces causing extinction and the rise of the initially rare OTUs is consistent with the action of forces causing immigration. These two processes can be deterministic or stochastic and, in light of the expected, dramatic effect on AMF community composition of the emergence and growth of the sorghum monoculture, determinism would seem the more likely explanation. Similarly, other factors argue against chance as the dominant force, including the paucity of rare OTUs in our communities (Fig. S3–S4), which minimizes the number of OTUs most susceptible to stochastic extinction [64], and the similarity in AMF community composition throughout the sorghum field (Fig. 1g–i), which limits the local pool of potential, stochastic immigrants.

The emergence of 13 significant indicator OTUs (Table S4) by the final time period, TP17, raises the question of coexistence of multiple species during succession. The creation of distinct niches by a developing host plant would favor coexistence of dissimilar species that could avoid competition by exploiting divergent niches (i.e., stabilizing niche differentiation, a process consistent with phylogenetic overdispersion) [65]. Conversely, the expansion of the same niche, as expected of a growing sorghum crop, would facilitate the immigration and coexistence of species adapted to the same environment. Successful immigrant taxa would be expected to show equal fitness in this expanding niche and, assuming that fitness traits are phylogenetically conserved, exhibit a phylogenetic underdispersion due to evolutionary relatedness [65]. We find a phylogenetic underdispersion of indicator AMF in the genera *Rhizophagus* and *Glomus* at TP17 (Table S4) in roots, rhizosphere, and soil (Fig. 4d–f) based calculation of the net relatedness index (NRI) from an ITS2 phylogeny (Fig. S2). The lack of significant phylogenetic underdispersion early in the season (Fig. 4d–f), indicative of stochastic community assembly, is consistent with our having planted sorghum in a fallowed field that was previously planted to oats and having no previous exposure to sorghum. Development of underdispersion, indicative of phylogenetic similarity of AMF community members, later in the season supports coexistence by equalizing fitness, likely due to the expanding niche, rather than avoiding competition by exploiting niche differences. A similar shift from initially random to significant phylogenetic relatedness has been reported for AMF communities of crop plants characterized by SSU OTUs in four soil samples taken over 9 weeks, but not for root samples, where the pattern was nonlinear over time [30]. The interpretation of phylogenetic underdisperson with equalizing fitness similarity relies on the phylogenetic conservation of traits [66], but evidence of specific, adaptive traits in AMF remains rare [67, 68].

### Initial density-dependent AMF population demography

**Fig. 6:**
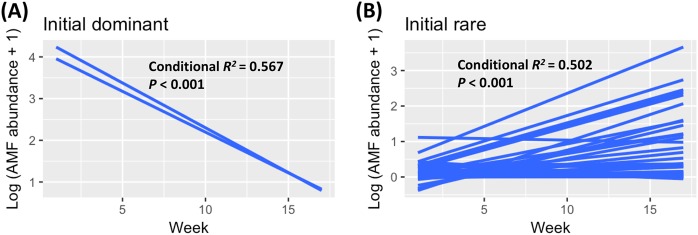
Steep (**a**) decline of initially dominant OTUs and (**b**) rise of at least 13 initially rare OTUs. Relationships between time and AMF OTU abundances were explored by linear mixed-effects models, including random effects of AMF identity. The conditional *R*^*2*^ calculated here can be interpreted as the variance explained by the mixed-effects models. Analyses in (**a**, **b**) treat sequence data as counts rarefied among AMF fungi and are nearly identical to analyses treating data as counts rarefied among all fungi or treating data as compositional (Fig. S7)

A role for population density in the decline of initially dominant OTUs and the rise of initially rare OTUs is suggested from our data, which document a decrease in relative abundance of the two OTUs dominant at TP01 (*Rhizophagus*_51, *Claroideoglomus*_70), and an increase in relative abundance of 13 OTUs rare at the same initial time period (Fig. 2b, c). In line with these observation, time is significantly negatively correlated with initial dominant OTUs and positively correlated with initial rare OTUs, as detected by linear mixed-effect modeling of all three datasets, whether rarefied for AMF reads, for all fungal reads, or not rarified and transformed by the CLR method (Fig. 6; Fig. S7). In the case of the two initially dominant OTUs whose relative abundance declined, they may have experienced a fitness disadvantage associated with high population density and their decline would be the result of competitive exclusion of species due to a disadvantage in fitness as compared to the rest of the community. Conversely, the population increases seen in the 13 initially rare OTUs may have been due to a fitness advantage at low population density, the magnitude of which would decrease as their populations grew [64]. Our results echo the only other studies to report replacement over time of dominant AMF OTUs [69, 70], in which the authors used SSU OTUs and five years of annual sampling to show that AMF OTUs dominant in newly germinated seedlings were almost entirely replaced by previously rare types; however, with few samples and broad OTU recognition, they were unable to correlate the replacement with population density [69, 70].

The negative density-dependent population growth observed here is explained in plant communities by two mechanisms, resource partitioning and escape from natural enemies [65]. Resource partitioning posits that different species either use different resources or partition the use of shared, limited resources [71]. As a result, species with large populations should experience limited population growth due to strong intraspecific competition, whereas species with small populations should experience high population growth due to the lack of intraspecific competition. However, support for the partitioning mechanism is not seen in the case of the six, closely related *Rhizophagus* OTUs (Table S4) that were shown, above, to be similar enough in fitness to avoid competitive exclusion and, therefore, too similar to occupy different niche spaces. Neither does partition theory appear to explain the inability of *Rhizophagus* OTU 51 to maintain population size in the final time period, likely due to competitive exclusion, because this process would not be expected to occur with effective partitioning [71]. Under the mechanism of natural enemy escape, species with large populations experience limited population growth rate due to the attraction and accumulation of more specific predators and pathogens, whereas species with small population experience high population growth rates due to the escape from host-specific natural enemies [65]. Alas, we do not have any data on predators and pathogens of AMF from our study, although these organisms must exist [72, 73].

The negative, density-dependent population growth seen for at least 13, initially rare, OTUs, indicates that populations of these AMF are able to increase in size while co-occurring with stable populations of other species. This invasibilty, together with the facts that these fungi live at the same trophic level and inhabit the same roots (Figs. 2 and 6; Table S4), suggests that these representatives of two AMF genera, *Rhizophagus* and *Glomus*, not only co-occur, but also co-exist [74]. These 13 AMF OTUs represent the first microbes where negative density-population growth in support of co-existence has been demonstrated over a long period [74], but further research will be needed to determine which phenotypic trade-offs may be associated with co-existence, such as, aspects of colonization and life-history strategy, differential interaction with host plants, peers and antagonists, and variation in adaptation to features of the abiotic environmental.

### Different AMF in root, rhizosphere, and soil

**Fig. 7:**
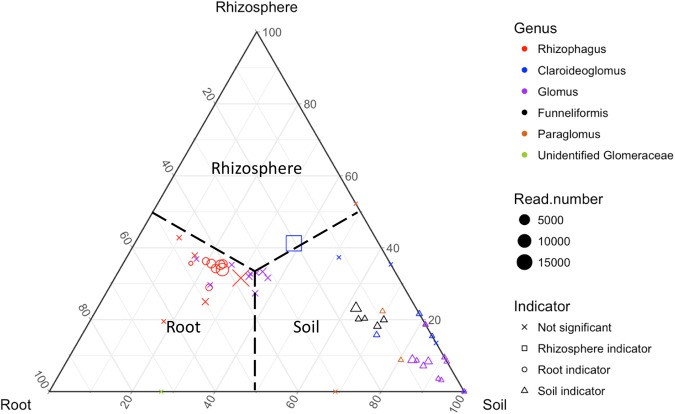
Ternary plot demonstrating the distribution of arbuscular mycorrhizal fungal (AMF) operational taxonomic units (OTUs) recovered from root, rhizosphere and soil. Note a bias toward roots for *Rhizophagus* OTUs, toward rhizosphere for a Clariodeoglomus OTU, and toward soil for *Glomus, Claroideglomus, Funneliformis* and *Paraglomus* OTUs

AMF are obligately dependent upon carbon from the roots of plants, so we expected that the AMF communities of the rhizosphere and soil would follow those seen in the root. This pattern was evidenced by similar trends for AMF in roots, soil, and rhizosphere in terms of temporal distance and succession, geographic distance, richness, and phylogenetic relatedness, as mentioned above. For example, the lag in response to nutrients provided by sorghum from roots to soil could be seen in the percentage of total fungal reads attributable to AMF, which peaked at TP02 in root, but peaked at TP04 in soil (Fig. 2c). Our data also suggest that different AMF species display different proportions of their thalli across the compartments of root, rhizosphere, and soil. In roots, six *Rhizophagus* OTUs were more commonly detected than in other compartments and, when detected, were more abundant (*indval* = 0.054 –0.399, *P* < 0.05; Table S5; Fig. 7). In rhizosphere, one *Claroideoglomus* OTU (*indval* = 0.419, *P* < 0.001) was more common and abundant than in other compartments (Table S5; Fig. 7). In soil, five *Funneliformis* OTUs (*indval* = 0.103–0.520, *P* < 0.01), three *Claroideoglomus* (*indval* = 0.051–0.167, *P* < 0.01), two *Paraglomus* (*indval* = 0.047–0.144, *P* < 0.05) and 11 *Glomus* (*indval* = 0.062–0.566, *P* < 0.01) were more common and abundant (Table S5; Fig. 7) than in other compartments. These results are consistent with observations that *Rhizophagus* species form abundant spores in the roots of vascular plants, whereas *Funneliformis* species form spores in the soil [75]. This variation in AMF morphology in nature also raises the possibility that AMF morphology could change over time, thereby adding variation associated with function [1] to studies of community composition.

## Conclusion

Our ability to demonstrate a strong signal of succession in AMF community composition over the sorghum growing season almost certainly rests on our choice of an experimental system with fewer variables than other studies (Table S1) as well as characterization of OTUs by ITS2, which recognizes species-level taxa [2, 10, 11, 31, 32]. Treating DNA sequence data as counts or as compositional showed no loss of statistical significance of results. Our approach also found that succession in AMF communities of sorghum showed the pattern of turnover in addition to strong patterns of nestedness, as has been reported in other studies of AMF (Table S6). Unlike previous studies of AMF that reported stochastic assembly of AMF communities [76–78], we provide analyses that both immigration and extinction are deterministic in this relatively homogenous environment, based on the disappearance of initial dominant OTUs rather than rare OTUs and the homogeneity of AMF communities throughout the sorghum field, which fails to provide a pool of potential immigrants that might enter communities by chance. The increase in phylogenetic similarity (underdispersion) of the many OTUs that immigrated is consistent with equalized fitness rather than niche differentiation, as might be expected with one soil type and one host plant, although phylogenetic underdispersion of AMF has been reported for more complex systems (Table S7).

The energies supporting succession or, more broadly, temporal change in community composition, are different for the two partners of the arbuscular mycorrhizal symbiosis; the autotrophic plant community is supported by solar radiation and the heterotrophic AMF fungal community is supported by carbon fixed by the plant. For plants, the insolation inputs can be relatively consistent over the scale of plant community succession, but the energy provided to the AMF by the growing crop is clearly expanding with time. Therefore, when the abundance of specific AMF species declines during the season, the reduction can be a combination of both absolute reduction and, owing to the expanding resource provided by the plant, reduction relative to increasing abundance of other AMF species. Keeping this caveat about population density in mind, the disappearance of two initially dominant taxa suggests activity promoted by high population density, whereas the population growth of 13 immigrant OTUs suggests the opposite, activity promoted by low population density. For most of our ecological analyses, soil and rhizosphere showed the same results as our primary focus, sorghum roots. However, a difference in OTU abundance between roots on one hand and soil plus rhizosphere on the other correlates with the behavior of AMF genera, some of which live and sporulate predominately in the root and others that are known to sporulate prolifically outside the root, as has been reported in other studies of AMF (Table S8). Our study provides a foundation for more ambitious studies of AMF community ecology, where our simple experimental system would be enlarged to include diversity in hosts, soil, hydration and fertilization, with the eventual goal of effectively studying natural systems.

Due to our inability to cultivate AMF apart from plants, many ghosts have haunted our understanding of these fungi. Just as genomics is showing that the AMF life cycle is typical of other fungi in terms of sex [79] and nuclear variation within an individual [14, 15], mycobiome ecology is showing that AMF community assembly is not a matter of chance, but a process determined by biotic and abiotic factors [80]. The several studies noted above that also found patterns of nestedness (Table S6) and genetic similarity inferred from phylogenetic underdispersion (Table S7) suggest that there may be general rules for assembly of AMF communities that await discovery. The succession of AMF fungi seen here suggests that some AMF species could be more beneficial to sorghum production than others and that these species might be added to agricultural fields along with seeds or applied later in the season. Our approach would also be useful in monitoring the persistence and effects of such additions on the AMF communities of crop plants.

## Electronic supplementary material

Supplementary

Database S1

Database S2

## References

[1] Smith SE, Read DJ (2010). *Mycorrhizal symbiosis*, 3rd edn. Academic Press: London.

[2] Taylor JD, Helgason T, Öpik M (2017). Molecular Community Ecology of Arbuscular Mycorrhizal Fungi. Taylor JD, Helgason T, Öpik M (2017) Molecular community Ecology of Arbuscular Mycorrhizal Fungi. In: Dighton J, White JF, eds. The Fungal Community: its Organization and Role in the Ecosystem, 4th edn. CRC Press, pp 1–26.

[3] Horn S, Hempel S, Verbruggen E, Rillig MC, Caruso T (2017). Linking the community structure of arbuscular mycorrhizal fungi and plants: a story of interdependence[quest]. ISME J.

[4] Klironomos JN (2002). Feedback with soil biota contributes to plant rarity and invasiveness in communities. Nature.

[5] Rillig MC (2004). Arbuscular mycorrhizae and terrestrial ecosystem processes. Ecol Lett.

[6] Shi NN, Gao C, Zheng Y, Guo LD (2016). Arbuscular mycorrhizal fungus identity and diversity influence subtropical tree competition. Fungal Ecol.

[7] van der Heijden M, Klironomos J, Ursic M, Moutoglis P, Streitwolf-Engel R, Boller T (1998). Mycorrhizal fungal diversity determines plant biodiversity, ecosystem variability and productivity. Nature.

[8] Zobel M, Öpik M (2014). Plant and arbuscular mycorrhizal fungal (AMF) communities – which drives which?. J Veg Sci.

[9] Bever JD (1994). Feeback between plants and their soil communities in an old field. Community Ecol.

[10] Bruns TD, Corradi N, Redecker D, Taylor JW, Öpik M (2017). Glomeromycotina: what is a species and why should we care?. New Phytol.

[11] Bruns TD, Taylor JW (2016). Comment on “Global assessment of arbuscular mycorrhizal fungus diversity reveals very low endemism”. Science.

[12] Davison J, Moora M, Öpik M, Adholeya A, Ainsaar L, Bâ A (2015). Global assessment of arbuscular mycorrhizal fungus diversity reveals very low endemism. Science.

[13] Opik M., Davison J., Moora M., Partel M., Zobel M. (2016). Response to Comment on "Global assessment of arbuscular mycorrhizal fungus diversity reveals very low endemism". Science.

[14] Ropars J, Corradi N (2015). Homokaryotic vs heterokaryotic mycelium in arbuscular mycorrhizal fungi: different techniques, different results?. New Phytol.

[15] Ropars J, Toro KS, Noel J, Pelin A, Charron P, Farinelli L (2016). Evidence for the sexual origin of heterokaryosis in arbuscular mycorrhizal fungi. Nat Microbiol.

[16] Thiéry O, Vasar M, Jairus T, Davison J, Roux C, Kivistik PA (2016). Sequence variation in nuclear ribosomal small subunit, internal transcribed spacer and large subunit regions of Rhizophagus irregularis and Gigaspora margarita is high and isolate-dependent. Mol Ecol.

[17] Johnson EA (1979). Succession an unfinished revolution. Ecology.

[18] Bruelheide H, Böhnke M, Both S, Fang T, Assmann T, Baruffol M (2011). Community assembly during secondary forest succession in a Chinese subtropical forest. Ecol Monogr.

[19] Johnson EA, Miyanishi K (2008). Testing the assumptions of chronosequences in succession. Ecol Lett.

[20] Walker LR, Wardle DA, Bardgett RD, Clarkson BD (2010). The use of chronosequences in studies of ecological succession and soil development. J Ecol.

[21] Datta MS, Sliwerska E, Gore J, Polz MF, Cordero OX (2016). Microbial interactions lead to rapid micro-scale successions on model marine particles. Nat Commun.

[22] Fierer N, Nemergut D, Knight R, Craine JM (2010). Changes through time: integrating microorganisms into the study of succession. Res Microbiol.

[23] Gao C, Zhang Y, Shi N, Zheng Y, Chen L, Wubet T (2015). Community assembly of ectomycorrhizal fungi along a subtropical secondary forest succession. New Phytol.

[24] Ortiz-Álvarez R, Fierer N, de los Ríos A, Casamayor EO, Barberán A (2018). Consistent changes in the taxonomic structure and functional attributes of bacterial communities during primary succession. ISME J.

[25] Wolfe JE, Button JE, Santarelli M, Dutton RJ (2014). Cheese rind communities provide tractable systems for in situ and in vitro studies of microbial diversity. Cell.

[26] Xiao C, Lu Z-M, Zhang X-J, Wang S-T, Ao L, Shen C-H et al. (2017). Bio-Heat Is a Key Environmental Driver Shaping the Microbial Community of Medium-Temperature Daqu. Appl Environ Microbiol. 83. 10.1128/AEM.01550-17 PMC5691423 28970223

[27] Bahram M, Peay KG, Tedersoo L (2015). Local-scale biogeography and spatiotemporal variability in communities of mycorrhizal fungi. New Phytol.

[28] Voříšková J, Brabcová V, Cajthaml T, Baldrian P (2014). Seasonal dynamics of fungal communities in a temperate oak forest soil. New Phytol.

[29] Han LL, Wang JT, Yang SH, Chen WF, Zhang LM, He JZ (2017). Temporal dynamics of fungal communities in soybean rhizosphere. J Soils Sediment.

[30] Bainard LD, Bainard JD, Hamel C, Gan Y (2014). Spatial and temporal structuring of arbuscular mycorrhizal communities is differentially influenced by abiotic factors and host crop in a semi-arid prairie agroecosystem. FEMS Microbiol Ecol.

[31] Kohout P, Sudová R, Janoušková M, Čtvrtlíková M, Hejda M, Pánková H (2014). Comparison of commonly used primer sets for evaluating arbuscular mycorrhizal fungal communities: Is there a universal solution?. Soil Biol Biochem.

[32] Schlaeppi K, Bender SF, Mascher F, Russo G, Patrignani A, Camenzind T (2016). High-resolution community profiling of arbuscular mycorrhizal fungi. New Phytol.

[33] Gao C, Shi NN, Chen L, Ji NN, Wu BW, Wang YL (2017). Relationships between soil fungal and woody plant assemblages differ between ridge and valley habitats in a subtropical mountain forest. New Phytol.

[34] Schoch CL, Seifert KA, Huhndorf S, Robert V, Spouge JL, Levesque CA (2012). Nuclear ribosomal internal transcribed spacer (ITS) region as a universal DNA barcode marker for Fungi. Proc Natl Acad Sci USA.

[35] Talbot JM, Bruns TD, Taylor JW, Smith DP, Branco S, Glassman SI (2014). Endemism and functional convergence across the North American soil mycobiome. Proc Natl Acad Sci USA.

[36] Tedersoo L, Bahram M, Põlme S, Kõljalg U, Yorou NS, Wijesundera R (2014). Global diversity and geography of soil fungi. Science.

[37] Stockinger H, Kruger M, Schussler A (2010). DNA barcoding of arbuscular mycorrhizal fungi. New Phytol.

[38] Taylor DL, Walters WA, Lennon NJ, Bochicchio J, Krohn A, Caporaso JG (2016). Accurate estimation of fungal diversity and abundance through improved lineage-specific primers optimized for Illumina amplicon sequencing. Appl Environ Microbiol.

[39] Xu L, Naylor D, Dong Z, Simmons T, Pierroz G, Hixson KK (2018). Drought delays development of the sorghum root microbiome and enriches for monoderm bacteria. Proc Natl Acad Sci Usa.

[40] Fadrosh DW, Ma B, Gajer P, Sengamalay N, Ott S, Brotman RM (2014). An improved dual-indexing approach for multiplexed 16S rRNA gene sequencing on the Illumina MiSeq platform. Microbiome.

[41] Krüger M, Krüger C, Walker C, Stockinger H, Schüßler A (2012). Phylogenetic reference data for systematics and phylotaxonomy of arbuscular mycorrhizal fungi from phylum to species level. New Phytol.

[42] Andrews S (2010). FastQC: a quality control tool for high throughput sequence data.

[43] Edgar RC (2013). UPARSE: highly accurate OTU sequences from microbial amplicon reads. Nat Methods.

[44] Martin M (2011). Cutadapt removes adapter sequences from high-throughput sequencing reads. EMBnet J.

[45] Kõljalg U, Nilsson RH, Abarenkov K, Tedersoo L, Taylor AFS, Bahram M (2013). Towards a unified paradigm for sequence-based identification of fungi. Mol Ecol.

[46] Katoh K, Standley DM (2013). MAFFT multiple sequence alignment software version 7: improvements in performance and usability. Mol Biol Evol.

[47] Tamura K, Stecher G, Peterson D, Filipski A, Kumar S (2013). MEGA6: molecular evolutionary genetics analysis version 6.0. Mol Biol Evol.

[48] Gloor GB, Macklaim JM, Pawlowsky-Glahn V, Egozcue JJ (2017). Microbiome datasets are compositional: and this is not optional. F Microbiol.

[49] Oksanen J, Blanchet FG, Kindt R, Legendre P, Minchin PR, O’Hara RB et al. (2013). Vegan: community ecology package. R package version 2.0-10.

[50] R Development Core Team. (2018). R: a Language and Environment for Statistical Computing.

[51] Palarea-Albaladejo J, Martín-Fernández JA (2015). zCompositions—R package for multivariate imputation of left-censored data under a compositional approach. Chemom Intell Lab Syst.

[52] Baselga A (2010). Partitioning the turnover and nestedness components of beta diversity. Glob Ecol Biogeogr.

[53] Kembel SW, Cowan PD, Helmus MR, Cornwell WK, Morlon H, Ackerly DD (2010). Picante: R tools for integrating phylogenies and ecology. Bioinformatics.

[54] De Boeck P, Bakkar M, Zwitser R, Nivard M, Hofman A, Tuerlinckx F (2011). The estimation of item response models with the lmer function from the lme4 package in R. J Stat Softw.

[55] Bartoń K (2013). MuMIn: Multi-model inference. R package version 1.9. 13. The Comprehensive R Archive Network (CRAN), Vienna, Austria. https://cran.r-project.org/web/packages/MuMIn/index.html.

[56] Arbuckle JL (2011). IBM® SPSS®Amos™20 User’s Guide.

[57] Anderson MJ, Walsh DCI (2013). PERMANOVA, ANOSIM, and the Mantel test in the face of heterogeneous dispersions: What null hypothesis are you testing?. Ecol Monogr.

[58] Paradis E, Claude J, Strimmer K (2004). APE: analyses of phylogenetics and evolution in R language. Bioinformatics.

[59] Baselga A, Orme CDL (2012). betapart: an R package for the study of beta diversity. Methods Ecol Evol.

[60] Krüger M, Stockinger H, Krüger C, Schüßler A (2009). DNA-based species level detection of Glomeromycota: one PCR primer set for all arbuscular mycorrhizal fungi. New Phytol.

[61] Lekberg Y, Gibbons SM, Rosendahl S (2014). Will different OTU delineation methods change interpretation of arbuscular mycorrhizal fungal community patterns?. New Phytol.

[62] Stockinger H, Walker C, Schüßler A (2009). ‘Glomus intraradices DAOM197198’, a model fungus in arbuscular mycorrhiza research, is not Glomus intraradices. New Phytol.

[63] García de León D, Moora M, Öpik M, Jairus T, Neuenkamp L, Vasar M (2016). Dispersal of arbuscular mycorrhizal fungi and plants during succession. Acta Oecol-Int J Ecol.

[64] Vellend M (2010). Conceptual synthesis in community ecology. Q Rev Biol.

[65] HilleRisLambers J, Adler PB, Harpole WS, Levine JM, Mayfield MM (2012). Rethinking community assembly through the lens of coexistence theory. Annu Rev Ecol Evol Syst.

[66] Kraft NJB, Cornwell WK, Webb CO, Ackerly DD (2007). Trait evolution, community assembly, and the phylogenetic structure of ecological communities. Am Nat.

[67] Koch AM, Antunes PM, Maherali H, Hart MM, Klironomos JN (2017). Evolutionary asymmetry in the arbuscular mycorrhizal symbiosis: conservatism in fungal morphology does not predict host plant growth. New Phytol.

[68] Powell JR, Parrent JL, Hart MM, Klironomos JN, Rillig MC, Maherali H (2009). Phylogenetic trait conservatism and the evolution of functional trade-offs in arbuscular mycorrhizal fungi. Proc R Soc Lond Ser B-Biol Sci.

[69] Husband R, Herre EA, Turner SL, Gallery R, Young JPW (2002). Molecular diversity of arbuscular mycorrhizal fungi and patterns of host association over time and space in a tropical forest. Mol Ecol.

[70] Husband R, Herre EA, Young JPW (2002). Temporal variation in the arbuscular mycorrhizal communities colonising seedlings in a tropical forest. FEMS Microbiol Ecol.

[71] Bruns TD (1995). Thoughts on the processes that maintain local species-diversity of ectomycorrhizal fungi. Plant Soil.

[72] Geisen S, Koller R, Hünninghaus M, Dumack K, Urich T, Bonkowski M (2016). The soil food web revisited: diverse and widespread mycophagous soil protists. Soil Biol Biochem.

[73] Purin S, Rillig MC (2008). Parasitism of arbuscular mycorrhizal fungi: reviewing the evidence. FEMS Microbiol Ecol.

[74] Siepielski AM, McPeek MA (2010). On the evidence for species coexistence: a critique of the coexistence program. Ecology.

[75] Schüßler A, Walker C (2010). The Glomeromycota: a species list with new families and new genera. Createspace: Gloucester.

[76] Caruso T, Hempel S, Powell JR, Barto EK, Rillig MC (2012). Compositional divergence and convergence in arbuscular mycorrhizal fungal communities. Ecology.

[77] Lekberg Y, Schnoor T, Kjoller R, Gibbons SM, Hansen LH, Al-Soud WA (2012). 454-sequencing reveals stochastic local reassembly and high disturbance tolerance within arbuscular mycorrhizal fungal communities. J Ecol.

[78] Powell JR, Bennett AE (2016). Unpredictable assembly of arbuscular mycorrhizal fungal communities. Pedobiologia.

[79] Tisserant E, Malbreil M, Kuo A, Kohler A, Symeonidi A, Balestrini R (2013). Genome of an arbuscular mycorrhizal fungus provides insight into the oldest plant symbiosis. Proc Natl Acad Sci.

[80] Valyi K, Mardhiah U, Rillig MC, Hempel S (2016). Community assembly and coexistence in communities of arbuscular mycorrhizal fungi. ISME J.

