# Supplementary material for: Strong succession in arbuscular mycorrhizal fungal communities
Source: ISME J. 2018 Aug 31;13(1):214–26. doi: 10.1038/s41396-018-0264-0 (PMC6298956; doi:10.1038/s41396-018-0264-0)
Supplement: Supplementary file 1 — Supplementary [file 41396_2018_264_MOESM1_ESM.pdf]

Supplementary methods

Experiment site

This experiment was conducted at the Kearney Agricultural Research and Extension (KARE) Center in Parlier, CA, USA (36.6008° N, 119.5109° W). KARE is located in the Central Valley, a semiarid zone with a mean annual temperature of 17.8°C and mean annual precipitation of 325 mm, almost all of which falls between November and April. During the course of our experiment (27th May to 28th September, 2016), no precipitation occurred; the daily minimum temperature ranged from 7.8 to 22.8°C, and the daily maximum temperature ranged from 22.8 to 40.5°C (http://ipm.ucanr.edu/WEATHER/index.html). Soils at KARE were plowed before seeding and are characterized as Hanford sandy loam with a silky substratum and pH 7.37.

Experiment design and sampling

Two sorghum [*Sorghum bicolor* (L.) Moench] cultivars with similar flowering times, RTx430 and BTx642, were planted in three, separate, 16 × 8 m^2 plots (each with ten rows) with 3 m borders between plots (Fig. S1). The seeds were sown into pre-watered fields and left unirrigated for two weeks, which is standard agricultural management practice for sorghum in the Central Valley. From the 3rd week until the final harvest, the plants were watered using drip irrigation with 80% of calculated evapotranspiration on a weekly basis.

The trial was planted on 27th May, 2016 and plants emergence was recorded on 1st June. Weekly samples of root, rhizosphere and soil were taken in 2016 on June 8, 15, 22, 29; July 6, 13, 20, 27; August 3, 10, 17, 24, 31; and September 7, 14, 21, 28. Between 10:00 and 14:00 of every sampling date, ten or more individual sorghum plants were removed from randomly chosen locations within one of the central eight rows in each plot. Both rhizosphere and root samples were taken from the pool of these ten individuals. Roots were removed from the ten plants, mixed

together, transferred to 50 ml tubes with detergent-phosphate buffer (6.33 $NaH_2PO_4•H_2O$ and 8.5 g Na_2HPO_4•anhydrous in 1 L water, autoclaved; cooled, 200µl Silwet-77 added; pre-cooled in ice-water mixture), and vortexed at full speed for 2 min. The roots were removed from the tube, the liquid-filled tube was saved, and the roots were transferred to a 200-ml plastic cup with phosphate buffer without detergent (6.33 $NaH_2PO_4•H_2O$ and 8.5 g Na_2HPO_4•anhydrous in 1 L water, autoclaved; pre-cooled in ice-water mixture), vortexed at full speed for 1 min twice, dried by clean paper towels, put into aluminum packet and frozen in liquid nitrogen. The saved, liquid-filled tube containing the rhizosphere was centrifuged at full speed for 3 min, the buffer discarded and the rhizosphere pellet frozen in liquid nitrogen. Simultaneously, soil at 6" depth was collected adjacent to the ten sampled plants using 6" soil collection tubes. Ten samples were mixed, transferred to a 50-ml centrifuge tube, and frozen in liquid nitrogen. Thus, a total of 312 samples were taken, which comprise 17 weekly samples of the two cultivars, and three compartments (root, rhizosphere and soil), all with three replicates, plus six soil samples collected prior to planting. The frozen root, rhizosphere and soil samples were transferred to dry ice and transported by 18:00 on the day of collection to laboratories at the University of California, Berkeley where they were stored at -80°C until grinding.

Molecular analysis

Root samples were ground, separately, with liquid nitrogen in a cryogenic grinder (6875D Freezer/Mill, SpexSamplePrep, Stanmore, UK), and root DNA was extracted from 0.2 g ground sample using the MoBio PowerSoil DNA kit (MoBio, Carlsbad, CA, USA) with all centrifugation conducted at 4°C. Rhizosphere and soil DNA was extracted from 0.2 g samples using the MoBio PowerSoil DNA kit (MoBio, Carlsbad, CA, USA) following the manufacture's protocol. DNA concentration was measured with a Qubit dsDNA HS kit (Life Technologies Inc., Gaithersburg,

MD, USA) and DNAs were adjusted to 5 ng/μl with ddH_2O. In preparation for Illumina Miseq sequencing of amplicons of the fungal internal transcribed spacer 2 (ITS2) region, PCR was performed on all samples using forward and reverse primers designed to contain a 29 (forward) or 25 (reverse) base linker, a 12 base barcode, a 29 (forward) or 34 (reverse) base pad, a 0-8 base heterogeneity spacer (Fadrosh et al 2014), and either the fungal ITS2 specific 5.8SFun primer or ITS4Fun primer (Taylor et al 2016) (Table S2). We used Lee Taylor's ITS2 primers(Taylor et al 2016) because the 5.8SFun and ITS4Fun matched well with all Glomeromycotina lineages when we matched the primers with the SSU-ITS-LSU alignment (Krüger et al 2012) (Database S1-S2). The 5.8Fun primer starts at the 2078th base of the SSU-ITS-LSU alignment (Database S1); and the ITS4Fun primer starts at the 3508th base of the reverse complementary of SSU-ITS-LSU alignment (Database S2). PCR amplification employed the one-step PCR method in the Gene Amplification PCR System (BioRad Laboratories Inc.) with initial denaturation at 96°C for 2 min, followed by 35 cycles of 94°C for 30 s, 58°C for 40 s and 72°C for 2 min, and a final extension at 72°C for 10 min. Each amplification was carried out in a 25 μl reaction mixture containing 10 μl 5PRIME HotMaster Mix (Eppendorf-5Prime, Gaithersburg, MD, USA), 2.5 μl forward primer, 2.5 μl reverse primer, 2 μl template DNA, and 8 μl nuclease-free water. Amplicon libraries were produced from a pool of three different PCRs. The yields of PCR products were measured using a Qubit dsDNA HS kit (Life Technologies Inc., Gaithersburg, MD, USA) and 200 ng of DNA from each of the 312 samples were randomly assigned to four different pools. The pooled products were purified using AMPure magnetic beads (Beckman Coulter Inc., Brea, CA, USA) following the manufacturer's instructions. Libraries were quality checked for concentration and amplicon size using the Agilent 2100 Bioanalyzer (Agilent Technologies, Santa Clara, CA, USA) at the Vincent J. Coates Genomics Sequencing Laboratory (GSL, University of California, Berkeley, CA, USA).

Pyrosequencing was performed on the Illumina Miseq PE300 sequencing platform (Illumina, Inc., CA, USA) at the GSL. All the raw sequences are deposited in Sequence Read Archive (raw data) with the accession codes: Bioproject PRJNA412410 Biosamples SAMN07711256 - SAMN07711567.

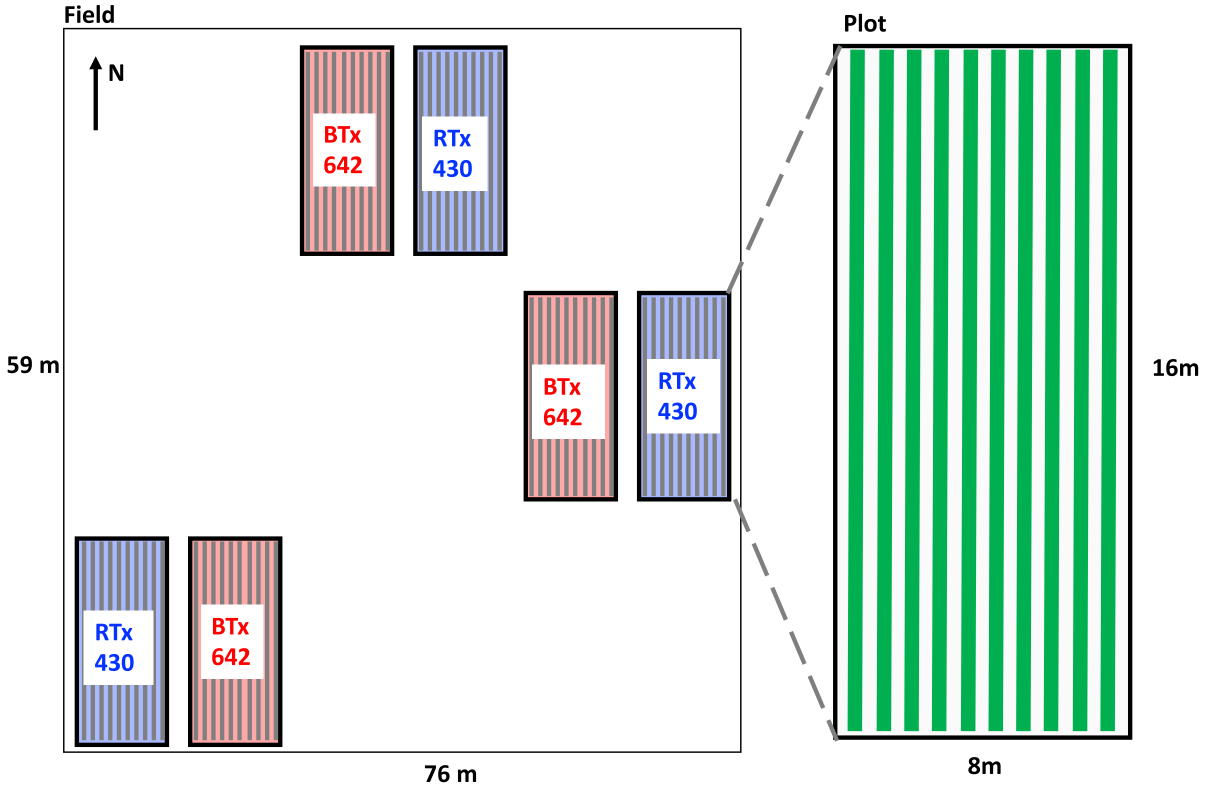

Fig. S1 Field layout of the six plots (16 x 8 m^2) of two sorghum cultivars (RTx430 and BTx642) in a field (76 x 59 m^2). Each plot consisted of ten rows of sorghum, each containing approximately 200 plants spaced 10cm apart. At each sampling time, plants were removed from randomly chosen locations within one of the central eight rows in each plot.

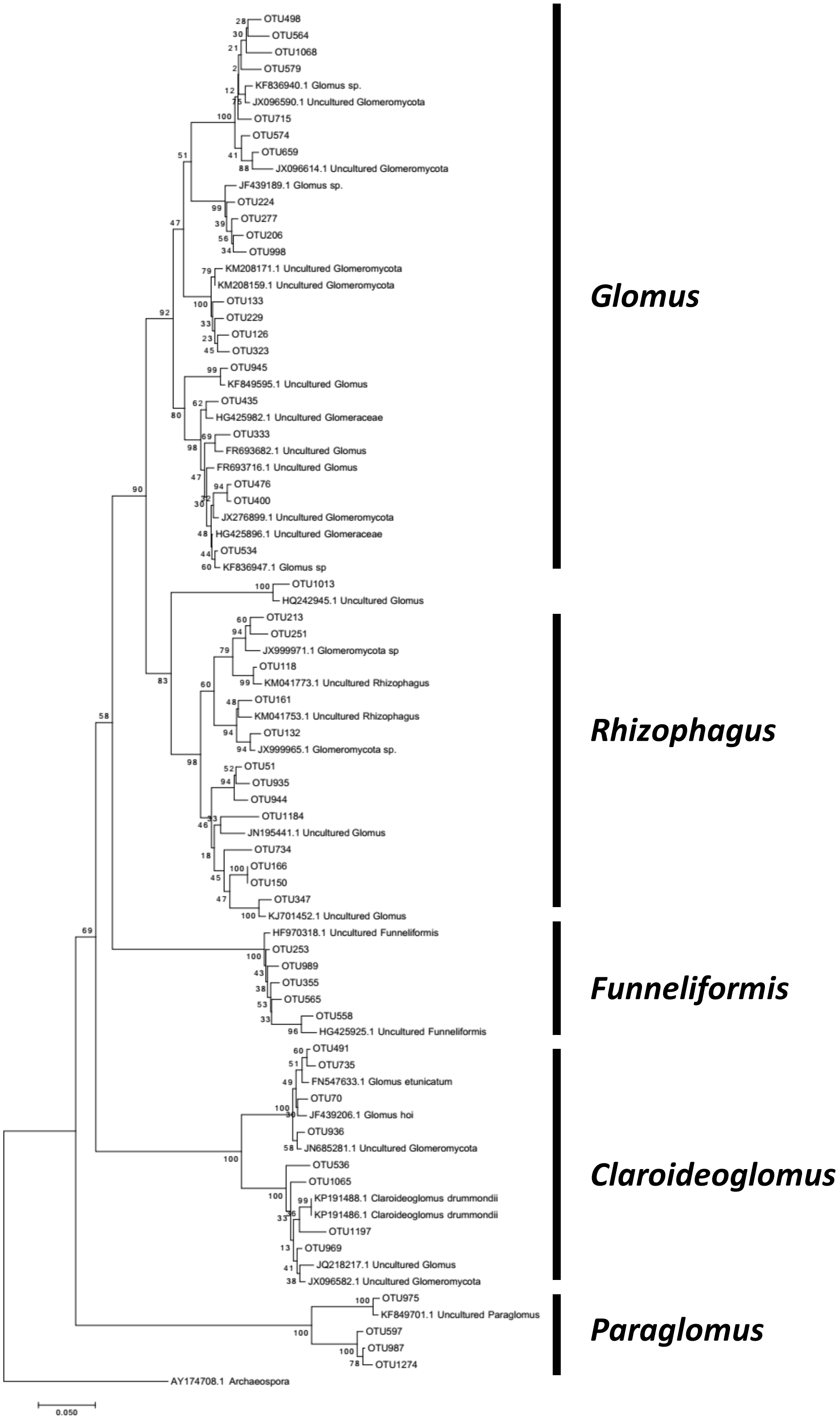

OTU498
OTU564
OTU1068
OTU579
KF836940.1 Glomus sp.
JX096590.1 Uncultured Glomeromycota
OTU715
OTU574
OTU659
JX096614.1 Uncultured Glomeromycota
JF439189.1 Glomus sp.
OTU224
OTU277
OTU206
OTU998
KM208171.1 Uncultured Glomeromycota
KM208159.1 Uncultured Glomeromycota
OTU133
OTU229
OTU126
OTU323
OTU945
KF849595.1 Uncultured Glomus
OTU435
HG425982.1 Uncultured Glomeraceae
OTU333
FR693682.1 Uncultured Glomus
FR693716.1 Uncultured Glomus
OTU476
OTU400
JX276899.1 Uncultured Glomeromycota
HG425896.1 Uncultured Glomeraceae
OTU534
KF836947.1 Glomus sp
OTU1013
HQ242945.1 Uncultured Glomus
OTU213
OTU251
JX999971.1 Glomeromycota sp
OTU118
KM041773.1 Uncultured Rhizophagus
OTU161
KM041753.1 Uncultured Rhizophagus
OTU132
JX999965.1 Glomeromycota sp.
OTU51
OTU935
OTU944
OTU1184
JN195441.1 Uncultured Glomus
OTU734
OTU166
OTU150
OTU347
KJ701452.1 Uncultured Glomus
HF970318.1 Uncultured Funneliformis
OTU253
OTU989
OTU355
OTU565
OTU558
HG425925.1 Uncultured Funneliformis
OTU491
OTU735
FN547633.1 Glomus etunicatum
OTU70
JF439206.1 Glomus hoi
OTU936
JN685281.1 Uncultured Glomeromycota
OTU536
OTU1065
KP191488.1 Claroideoglomus drummondii
KP191486.1 Claroideoglomus drummondii
OTU1197
OTU969
JQ218217.1 Uncultured Glomus
JX096582.1 Uncultured Glomeromycota
OTU975
KF849701.1 Uncultured Paraglomus
OTU597
OTU987
OTU1274
AY174708.1 Archaeospora
0.050
Glomus
Rhizophagus
Funneliformis
Claroideoglomus
Paraglomus

Fig. S2 Phylogenetic analysis of AMF ITS2 sequences of operational taxonomic units (OTUs) obtained in this study combined with named sequences from UNITE and NCBI. Generic names are applied to clades and the OTUs they contain based on named sequences that share the clade. Representative sequences of AMF OTUs were deposited in GenBank with the accession codes: MG008508 - MG008559. The phylogram is rooted with *Archaeospora* based on (BŁaszkowski et al 2006).

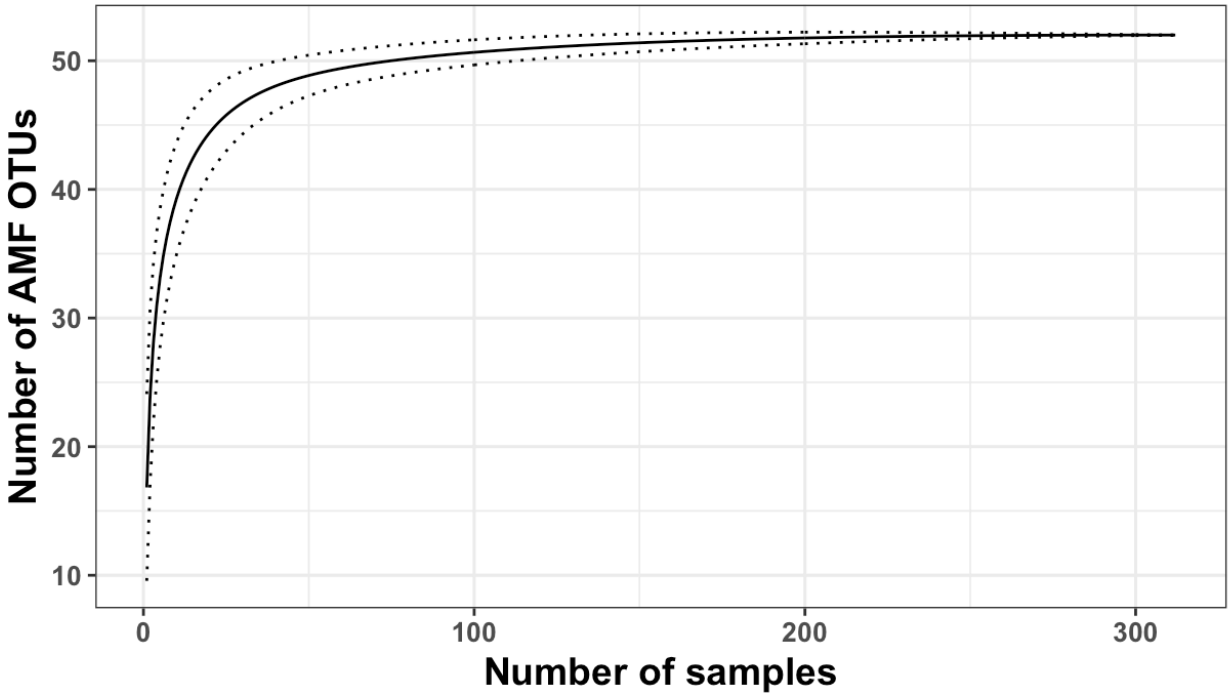

Fig. S3 The AMF species accumulation curve reaching a plateau of 48.86 ± 1.57 of 52 taxa after 50 samples.

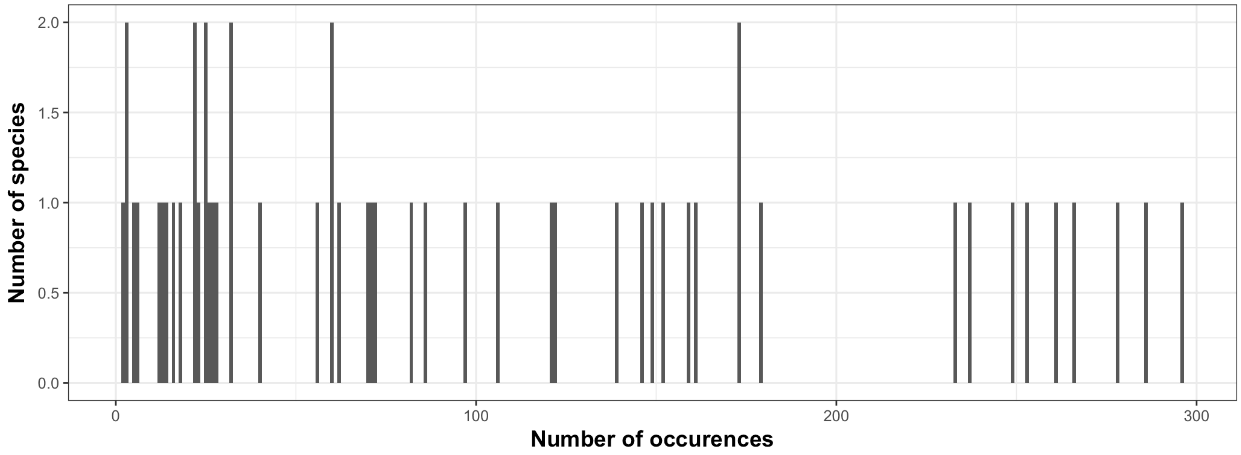

Fig. S4 The frequency of AMF OTUs found in as few as 2 to as many as 296 of all 312 communities sampled. Of the 52 AMF OTUs, only five were found in fewer than 10 samples and six were found in at least 250 samples.

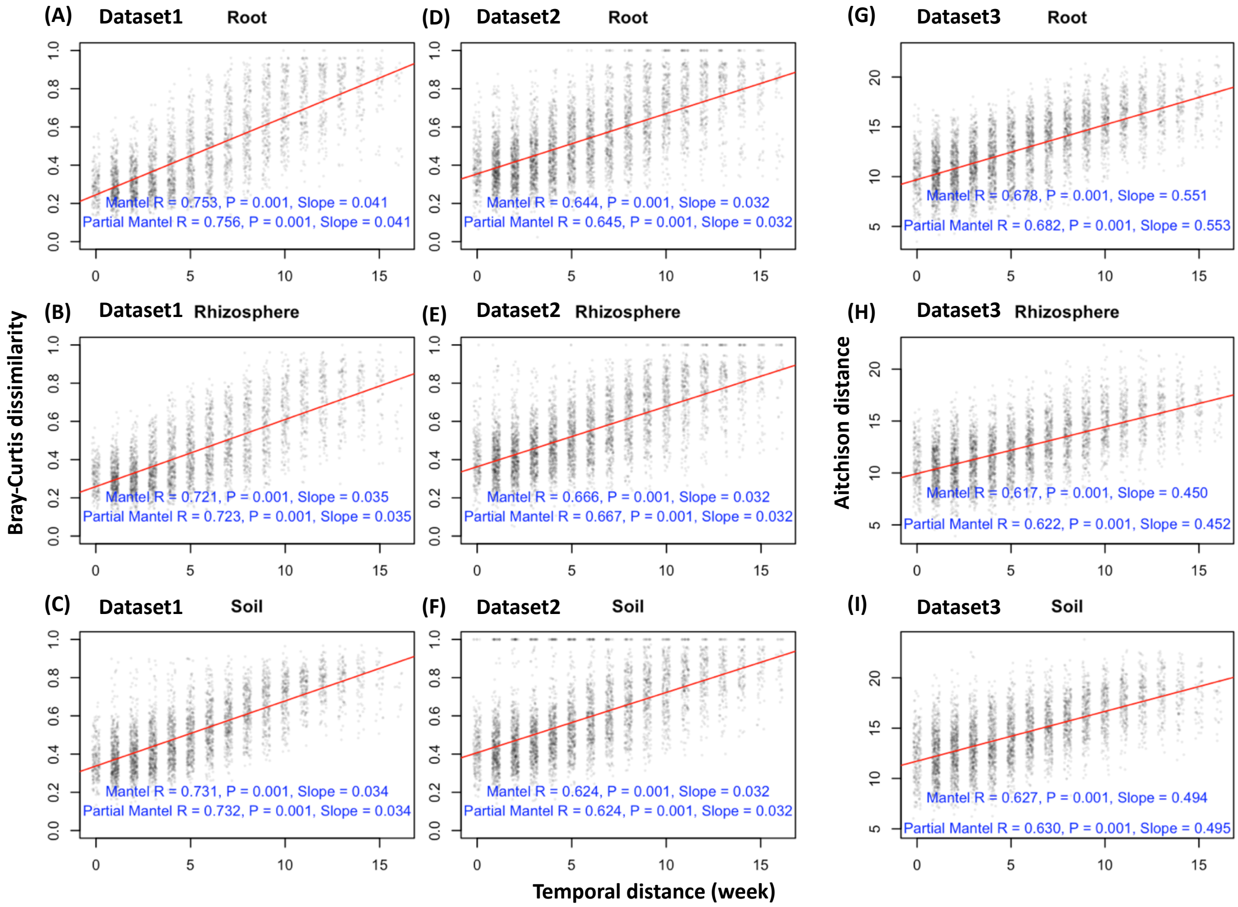

Fig. S5 Comparison of the correlation between AMF community change and temporal distance when treating the DNA sequence data as counts (A-F) or compositional (G-I) (Gloor et al 2017). Note that the result of strong succession is seen regardless of the analytical treatment. AMF datasets (A-C; See Figure 1) rarefied to equal AMF reads (dataset 1), (D-F) rarefied to equal fungal reads (dataset 2), and (G-I) transformed by the centered log-ratio method (dataset 3).

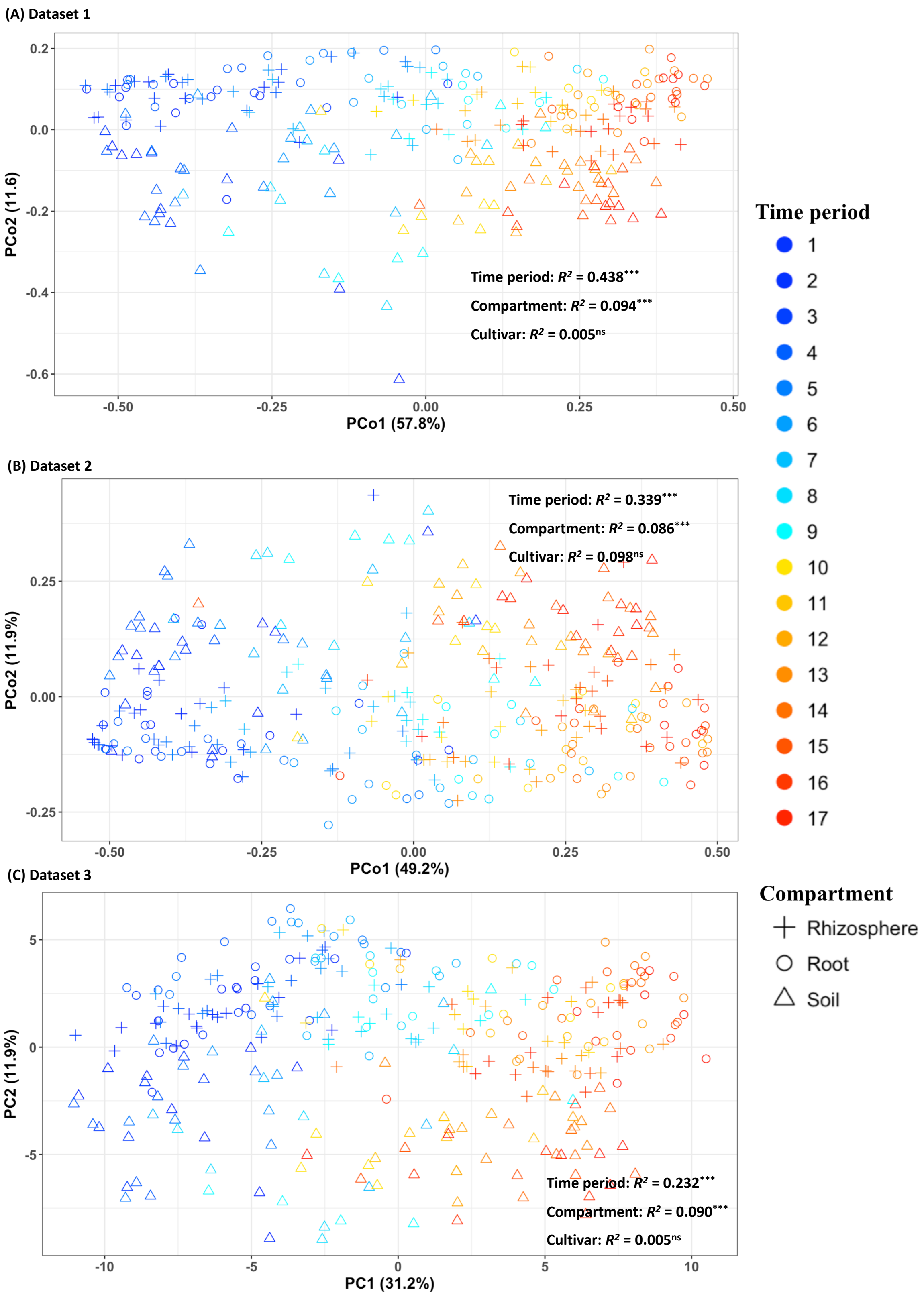
(A) Dataset 1
PCo1 (57.8%)
PCo2 (11.6)
Time period: R^2 = 0.438***
Compartment: R^2 = 0.094***
Cultivar: R^2 = 0.005ns
(B) Dataset 2
PCo1 (49.2%)
PCo2 (11.9%)
Time period: R^2 = 0.339***
Compartment: R^2 = 0.086***
Cultivar: R^2 = 0.098ns
(C) Dataset 3
PC1 (31.2%)
PC2 (11.9%)
Time period: R^2 = 0.232***
Compartment: R^2 = 0.090***
Cultivar: R^2 = 0.005ns
Time period
1
2
3
4
5
6
7
8
9
10
11
12
13
14
15
16
17
Compartment
Rhizosphere
Root
Soil

Fig. S6 Comparison of change in 17 weekly samples of composition of arbuscular mycorrhizal fungal communities of soil, rhizosphere and root when treating the DNA sequence data as counts (A, B) or as compositional (C). AMF datasets (A) rarefied to equal AMF reads (dataset 1) (see Figure 2A), (B) rarefied to equal fungal reads (dataset 2), and (C) transformed by the centered log-ratio method (dataset 3). As seen in Figure 2A, the strongest correlation is between community composition and time period, a result returned by all three methods of analysis. PCo: principal coordinate; PC: principal component.

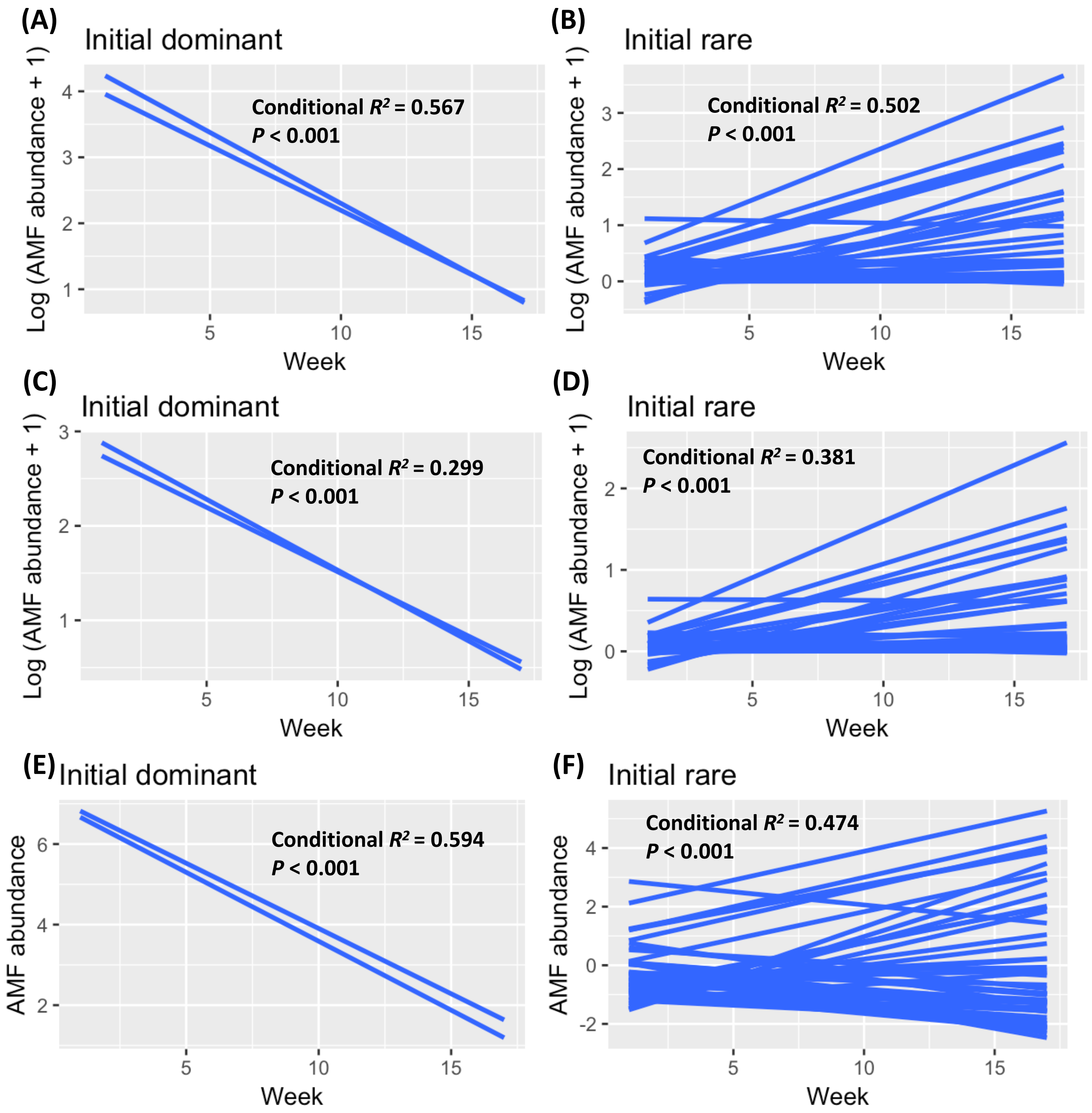

Fig. S7 Comparison of temporal change in initial dominant and initial rare arbuscular mycorrhizal fungal OTUs when treating the DNA sequence data as counts (A-D) or as compositional (E-F). AMF datasets (A-B, See Fig. 6) rarefied to equal AMF reads (dataset 1), (C-D) rarefied to equal fungal reads (dataset 2), and (E-F) transformed by the centered log-ratio method (dataset 3). Note

that the results are almost identical whether the data are treated as counts or considered to be compositional.

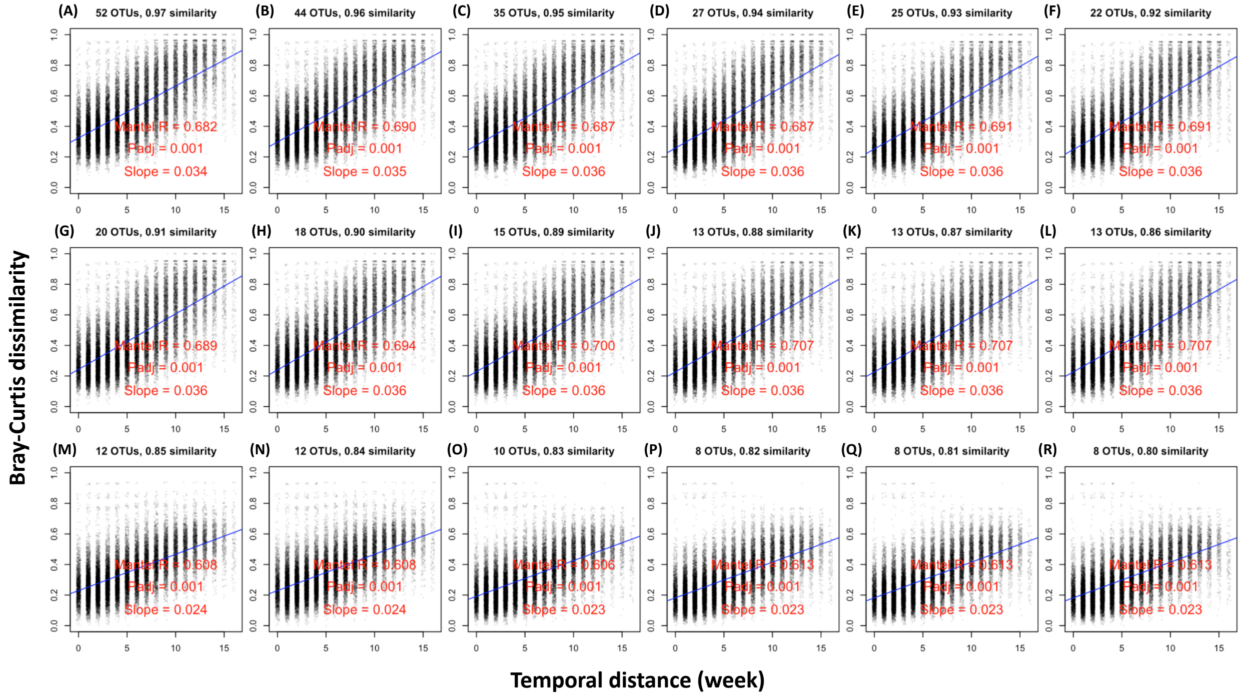

Fig. S8 Mantel correlation between temporal distance and Bray-Curtis dissimilarity of arbuscular mycorrhizal fungal (AMF) communities determined by reducing the sequence similarity used to delineate OTUs. Sequence similarities: (A) 97%, (B) 96%, (C) 95%, (D) 94%, (E) 93%, (F) 92%, (G) 91%, (H) 90%, (I) 89%, (J) 88%, (K) 87%, (L) 86%, (M) 85%, (N) 84%, (O) 83%, (P) 82%, (Q) 81% and (R) 80%. To improve visualization, we added a small amount of noise to the temporal distance and rendered the points transparent. The P value was adjusted by the Bonferroni method, to avoid the type-I error of multiple testing. **Note the sharp drop of association between community dissimilarity and temporal distance that occurred between the 86% cutoff (L) (slope = 0.036) and the 85% cutoff (M) (slope = 0.024). The slopes were stable prior to this point, 97% (A) to 86% (L), (slope = 0.034 -0.036) and after it, 85% (M) to 80% (R) (slope = 0.023 – 0.024).**

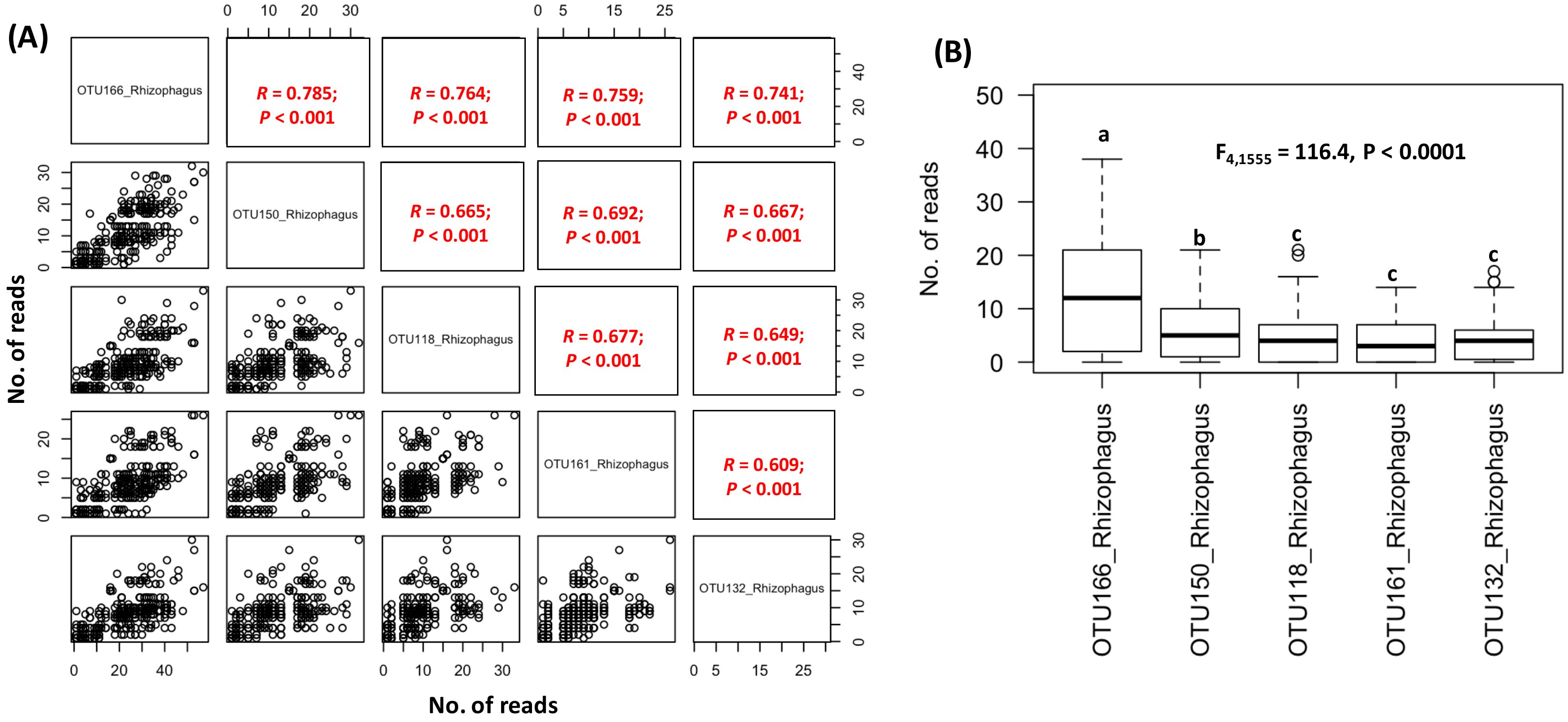

Fig. S9 Using the pattern and abundance of internal transcribed spacer 2 (ITS2) reads to identify possible cases of more than one ITS2 sequence in a single arbuscular mycorrhizal fungal (AMF) operational taxonomic unit (OTU). (A) Strong correlation (all $P < 0.001$) among abundance of five *Rhizophagus* OTUs. (B) Differences in the abundance among five *Rhizophagus* OTUs. Bars without shared letters indicate significant differences as determined by Tukey HSD. Based on their strongly correlated (A) and equalized abundance

(B), three OTUs (118, 161 and 132) were treated as a single species. Other two OTUs (166 and 150) were treated as different species because their abundances were unequal, despite their similar behavior.

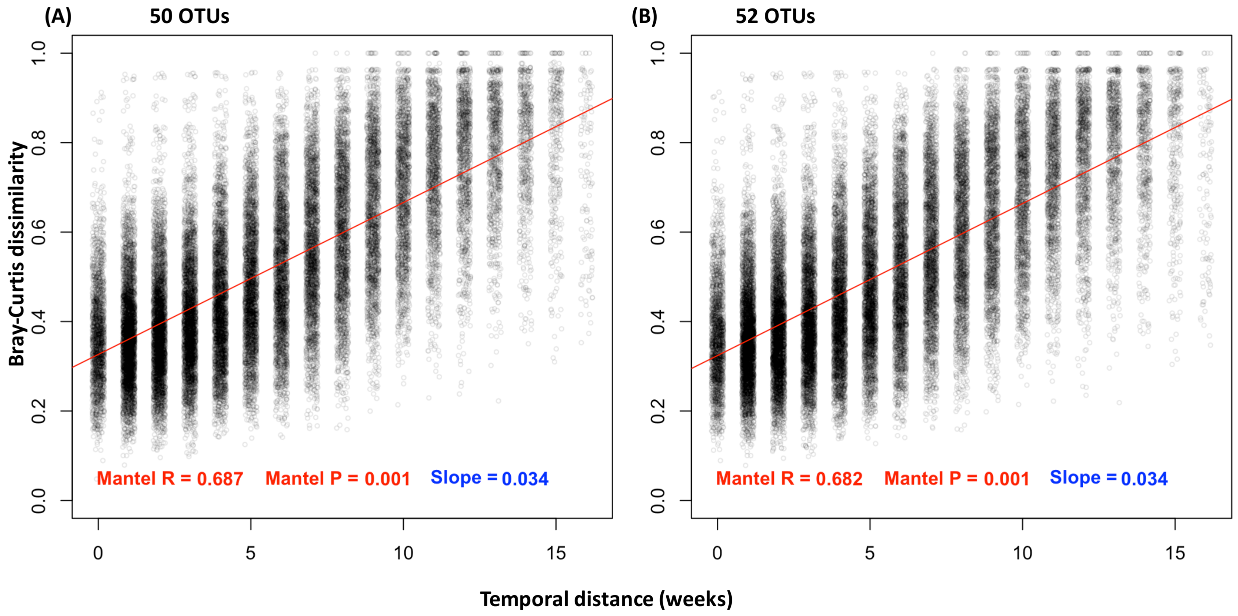

Fig. S10 Comparison of succession analyses with 50 or 52 arbuscular mycorrhizal fungal (AMF) operational taxonomic units (OTUs). No substantial difference in the succession pattern of AMF communities with three OTUs, i.e., 118, 161, 132 possibly belonging to one species, were (A) combined (50 OTUs dataset) or (B) not (52 OTUs dataset), as demonstrated by Mantel test between temporal distance and AMF Bray-Curtis dissimilarity. To improve visualization, we added a small amount of noise to the temporal distance and rendered the points transparent.

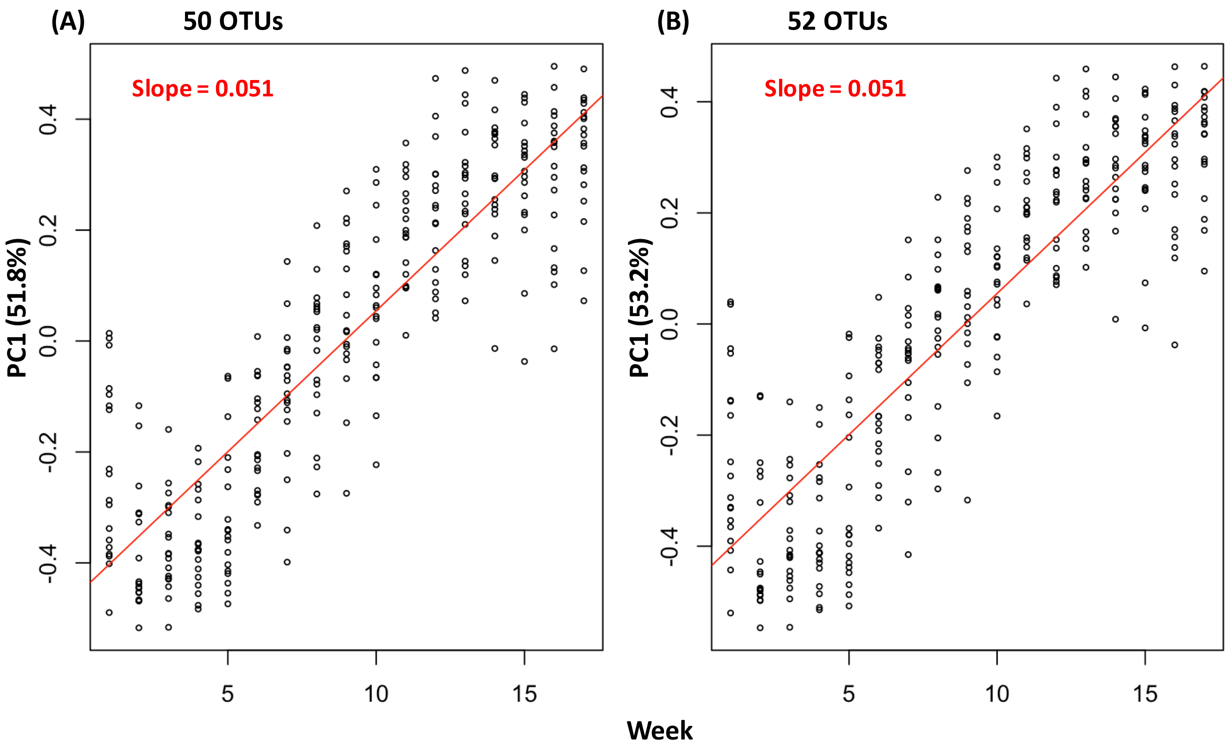

Fig. S11 Comparison of principal coordinate (PC) analyses with 50 or 52 arbuscular mycorrhizal fungal (AMF) operational taxonomic units (OTUs). No substantial difference in the temporal dynamic of AMF communities with three OTUs, i.e., 118, 161, 132 possibly belonging to one species, were (A) combined (50 OTUs dataset) or (B) not (52 OTUs dataset), as demonstrated by correlation between time and the first axis of PC analysis of AMF community

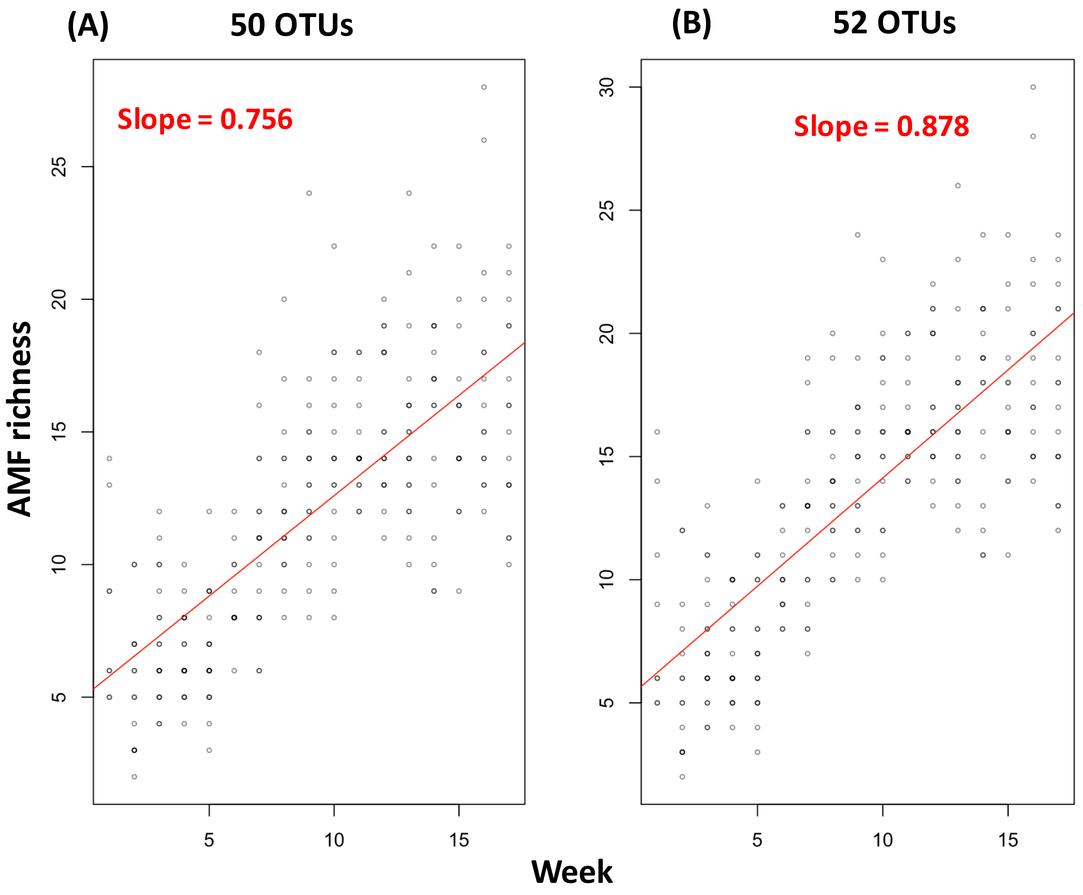

Fig. S12 Comparison of richness with 50 or 52 arbuscular mycorrhizal fungal (AMF) operational taxonomic units (OTUs). No substantial difference in the temporal dynamic of AMF richness with three OTUs, i.e., 118, 161, 132 possibly belonging to one species, were (A) combined (50 OTUs dataset) or (B) not (52 OTUs dataset), as demonstrated by correlation between time and the AMF richness.

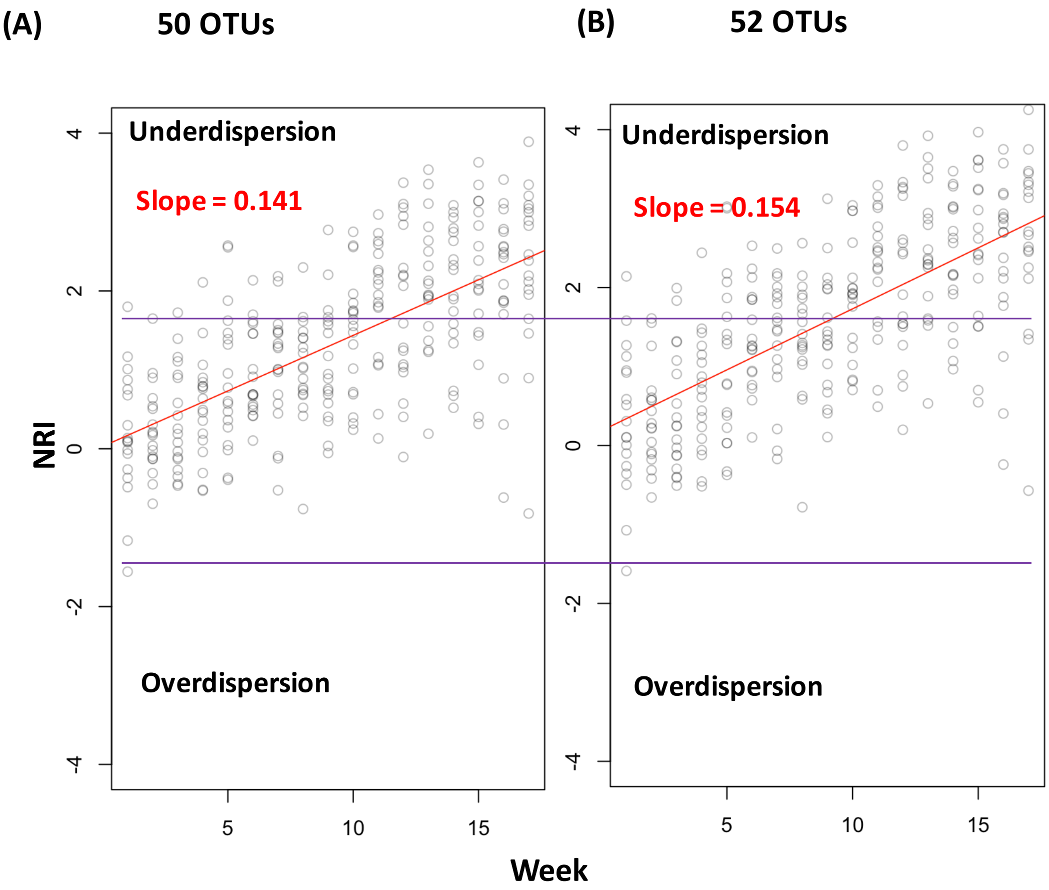

Fig. S13 Comparison of phylogenetic relatedness analyses with 50 or 52 arbuscular mycorrhizal fungal (AMF) operational taxonomic units (OTUs). No substantial difference in the temporal dynamic of net relatedness index (NRI) of AMF with three OTUs, i.e., 118, 161, 132 possibly belonging to one species, were (A) combined (50 OTUs dataset) or (B) not (52 OTUs dataset), as demonstrated by correlation between time and the AMF NRI. Note both datasets showed increases of NRI over time resulting in eventual, significant (above the upper purple horizontal line) underdispersion.

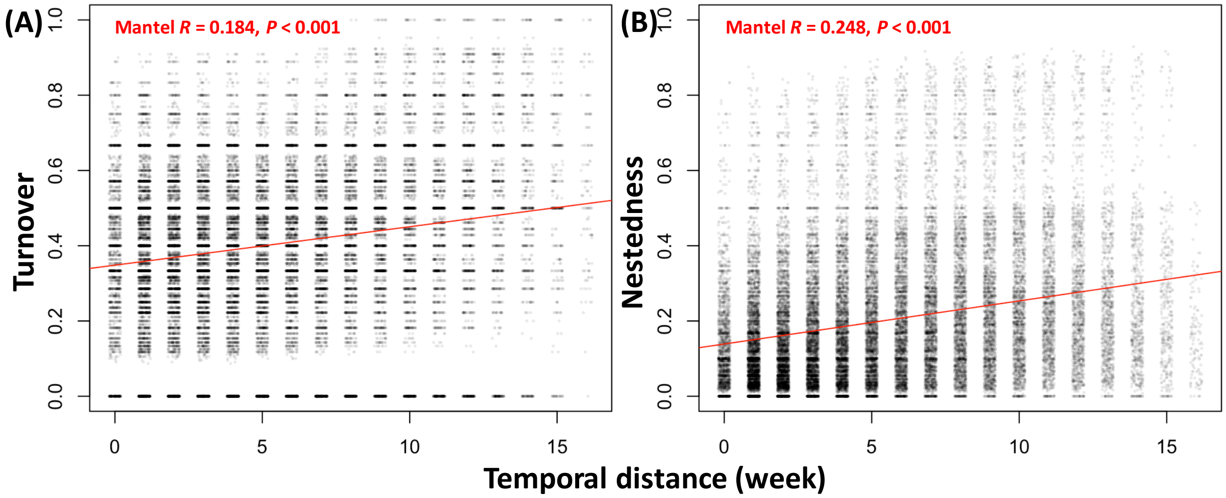

Fig. S14 Comparison of temporal distance and turnover and nestedness with 50 (Fig. 5) or 52 arbuscular mycorrhizal fungal (AMF) operational taxonomic units (OTUs). Both patterns of (A) turnover and (B) nestedness of AMF community over time are detected by Mantel test of the correlation between temporal distance and turnover and nestedness. To improve visualization, we added a small amount of noise to the temporal distance and rendered the points transparent. Note three OTUs, i.e., 118, 161, 132 possibly belonging to one species, were not combined in this analysis, and the results are not substantially different from those that were combined (Fig. 5).

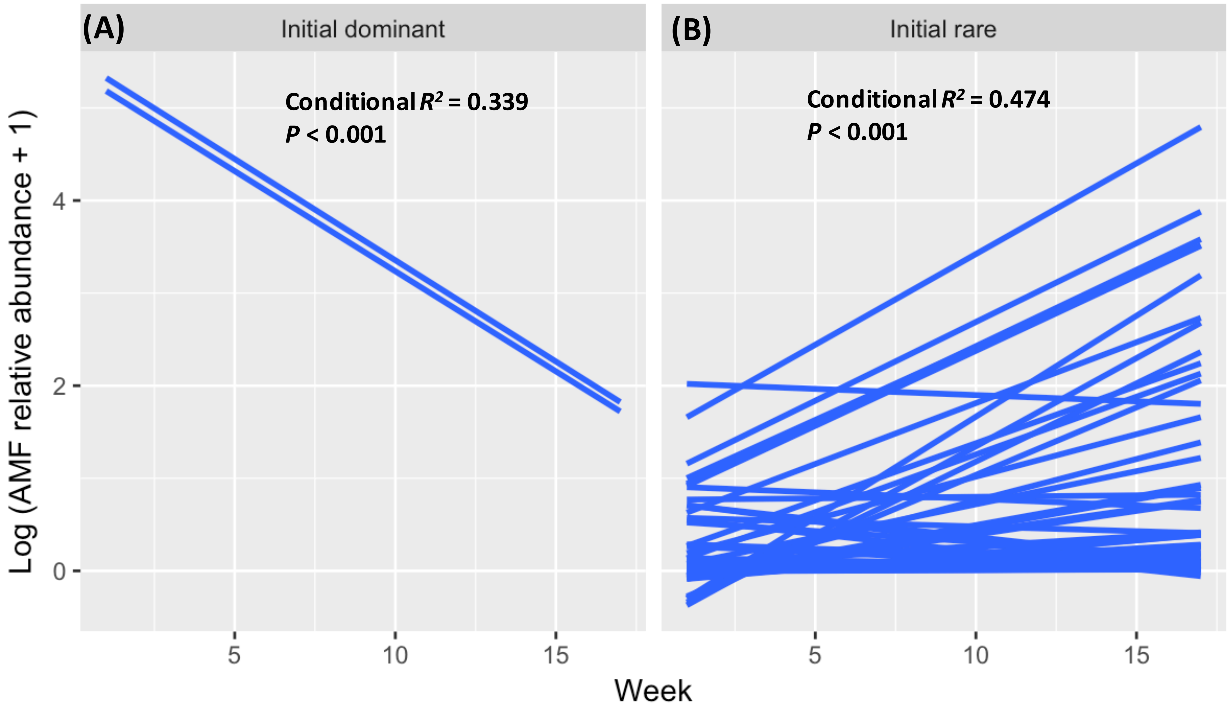

Fig. S15 Comparison of time and abundance analyses with 50 (Fig. 6) or 52 arbuscular mycorrhizal fungal (AMF) operational taxonomic units (OTUs). Relationships between time and abundance of initial dominant AMF OTUs, and initial rare AMF OTUs, as explored by linear mixed-effects models, including random effects of AMF identity. The conditional R^2 that can be interpreted as the variance explained by the mixed effect model was calculated. Note three OTUs, i.e. 118, 161, 132 possibly belonging to one species, were not combined in this analysis, and the results are not substantially different from those that were combined (Fig. 6)

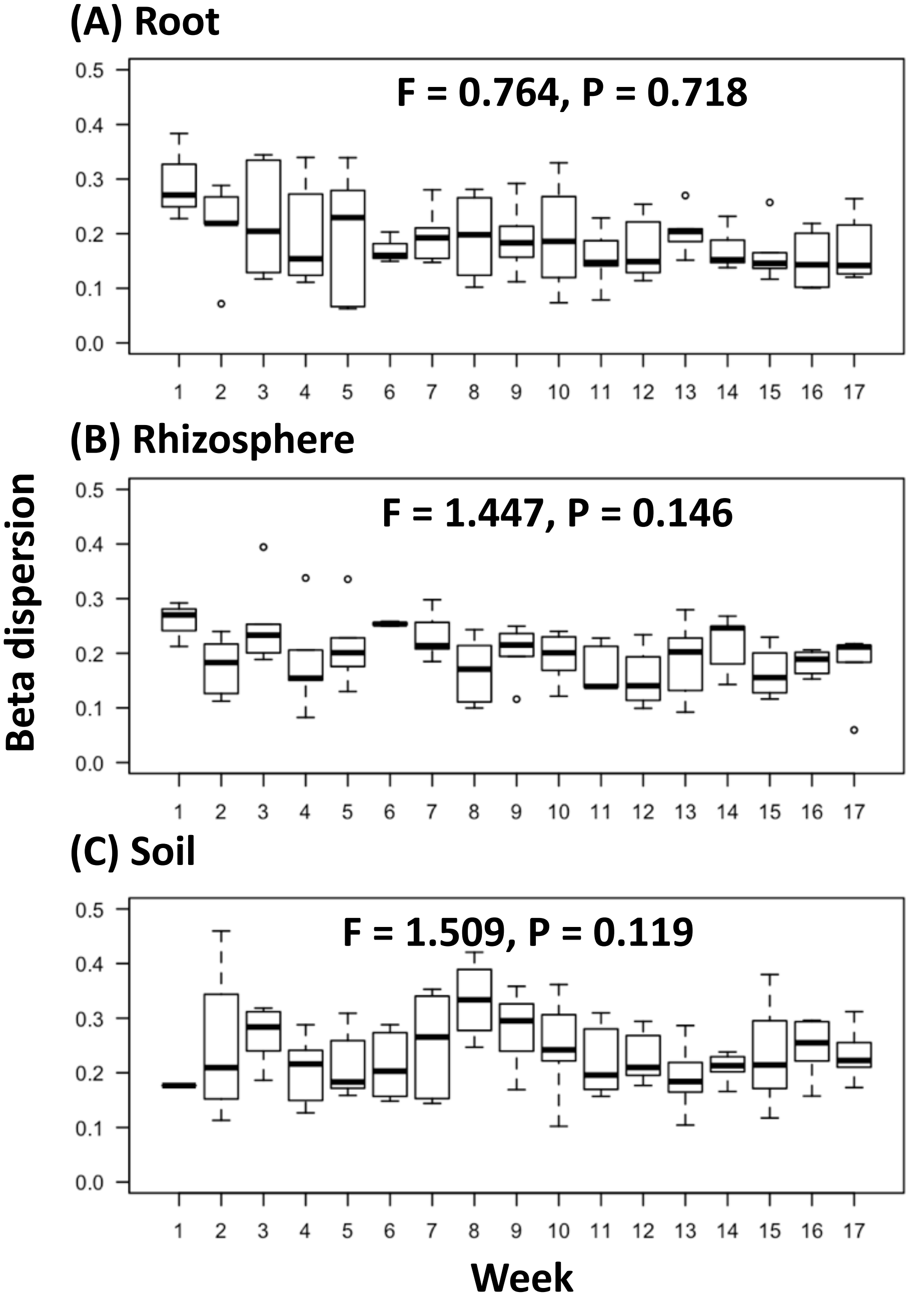

Fig. S16 Permuted beta-dispersion to test the community homogeneity of the arbuscular mycorrhizal fungal (AMF) communities within every sampling time period (TP) in (A) root, (B) rhizosphere, and (C) soil. Note the homogeneity of AMF community variances across all TPs in root, rhizosphere and soil, due to the lack of significant differences among TPs.

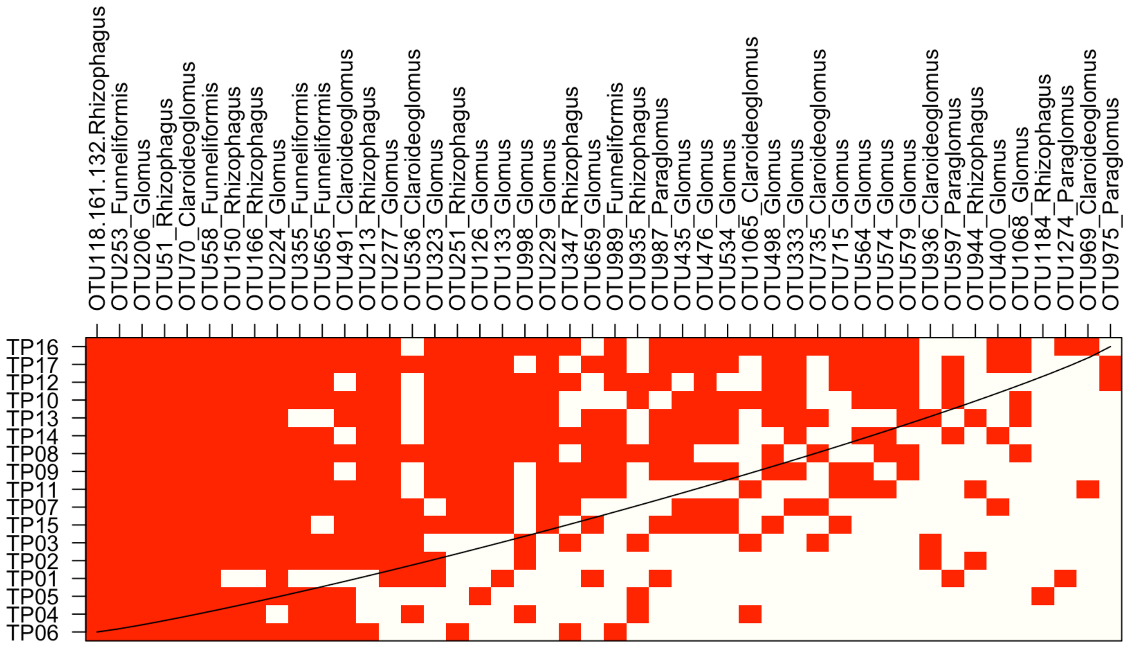

Fig. S17 Root samples showing a nested pattern of arbuscular mycorrhizal fungal (AMF) operational taxonomic units (OTUs) occurrence (in red) with time period (TP). Matrix of time × AMF is sorted to maximize nestedness by nestedtemp function in vegan package in R. The curved line shows isoclines of prefect nestedness.

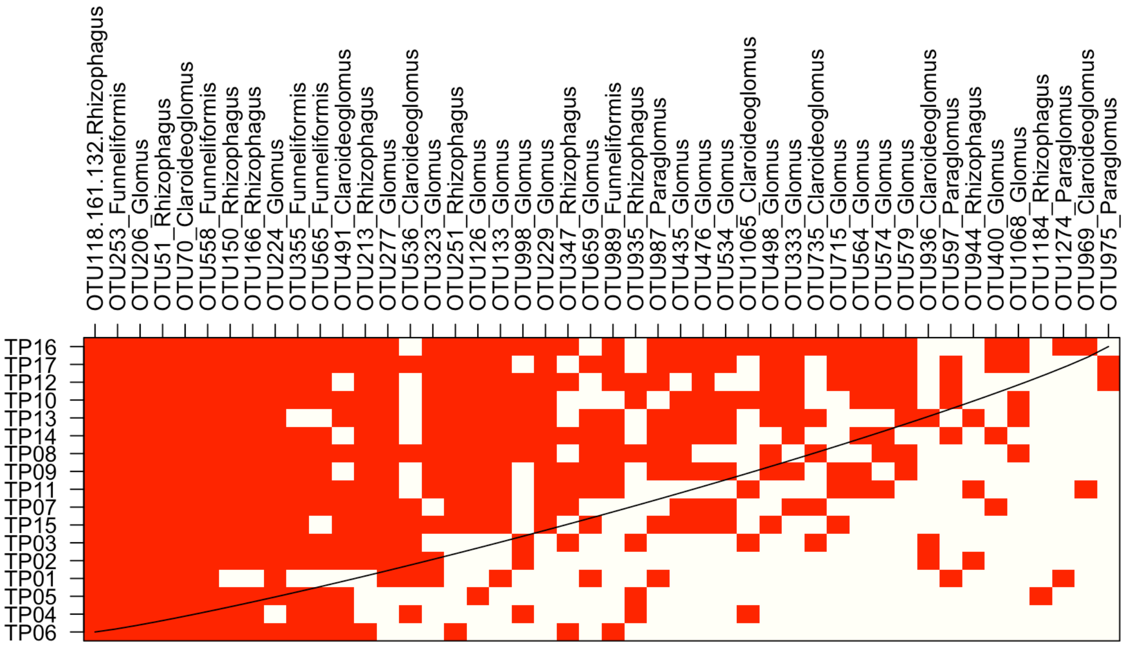

Fig. S18 Rhizosphere samples showing a nested pattern of arbuscular mycorrhizal fungal (AMF) operational taxonomic units (OTUs) occurrence (in red) with time period (TP). Matrix of time × AMF is sorted to maximize nestedness by nestedtemp function in vegan package in R. The curved line shows isoclines of prefect nestedness.

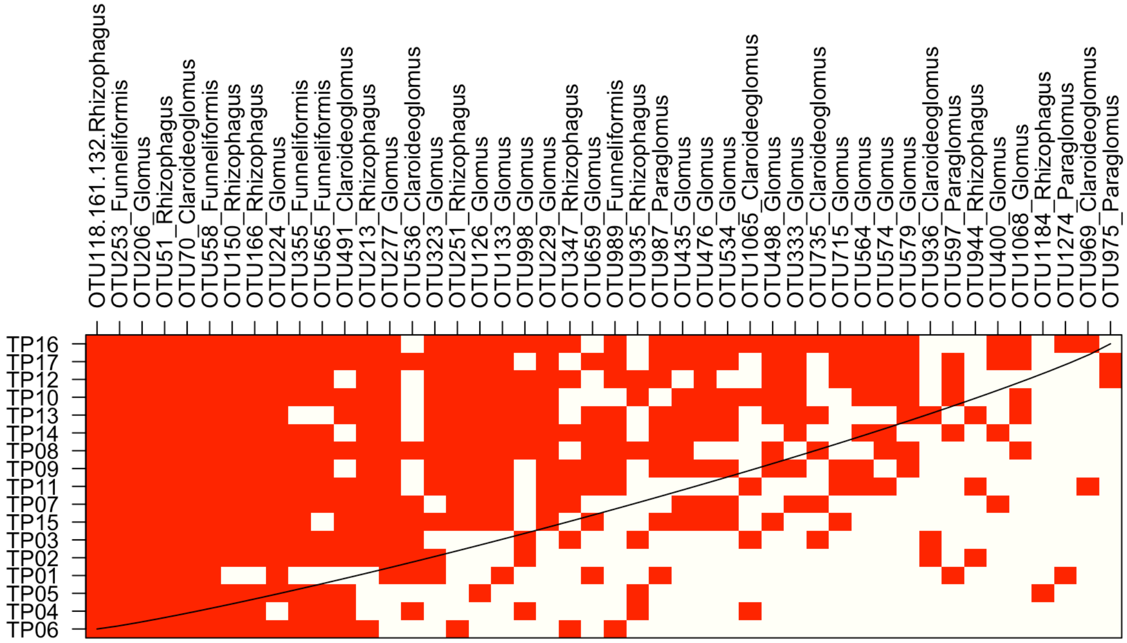

Fig. S19 Soil samples showing a nested pattern of arbuscular mycorrhizal fungal (AMF) operational taxonomic units (OTUs) occurrence (in red) with time period (TP). Matrix of time × AMF is sorted to maximize nestedness by nestedtemp function in vegan package in R. The curved line shows isoclines of prefect nestedness.

206

Table S1 A list of studies investigating the temporal dynamics of arbuscular mycorrhizal fungal (AMF) communities. Note succession of AMF communities often cannot be fully acknowledged due to (i) the lack of intensity sampling, (ii) poor AMF recognition resolution, and (iii) confounding influences by history, geography, climate and environments

Study	Ecosystem	AMF recognition	Sampling time	Sample type	Temporal change
(Bainard et al 2014)**	Farmed pea, lentil and wheat in temperate semiarid prairie	454 sequencing of AMF SSU	Four, tri-week samples	Root, soil	AMF richness, community composition, and net relatedness index changed by time.
(Yu et al 2012)	Pea grown in climate chamber	454 sequencing of fungal ITS	Vegetative growth, flowering, senescence	Root	Abundance of a *Glomus* AMF decreased, a *Paraglomus* AMF increased, and two *Glomus* AMF not changed with time
(Zeng et al 2014)	Maize farmed in subtropical China	tRFLP	Seedling, large ball, matured	Root, soil	AMF diversity and community composition were not influenced by sampling time
(Liu et al 2016)	Maize planted in temperate China	tRFLP	6-leaf, 13-leaf, kernel dough	Root	AMF community richness and composition changed by plant develop stage
(Bainard et al 2012)	Maize monocropped or intercropped by tree	tRFLP	Four, monthly samples (May-August)	Root	AMF community composition changed by time
(Turrini et al 2016)	Crop-maize succession	Sanger sequencing of AMF SSU	April and June	Root	AMF community composition differed between these two seasons
(Higo et al 2014, Higo et al 2015)	Soybean field with different winter rotation type in Japan	Sanger sequencing of AMF LSU	Five years (the flowering time of every year)	Root	AMF community composition differed by year
(Davison et al 2012)*	Temperate mixed forest	454 sequencing of AMF SSU	Four, monthly samples	Soil	AMF was not changed by time in ref. ; AMF community composition changed by time weakly, as demonstrated by reanalysis of ref. .

(Voříškov á et al 2014)**	Temperate oak woodland	454 sequencing of fungal ITS	Four, seasonal samples	Soil	AMF community composition changed by time, as demonstrated by reanalysis of ref. .
(Helgason et al 2014)*	Maples (eight spp.) in England garden	tRFLP	April, June, October	Root	Both AMF richness and composition affected by the three seasons
(López-García et al 2014)	Rosemary seedlings, in a mesocosm system	tRFLP	Every three months in two years	Root	AMF community composition was affected by season
(Varela-Cervero et al 2016)	Temperate forest, 5 tree species	tRFLP	Autumn, spring	Root Soil	AMF community composition was reported to be influenced by season in root, but not in soil.
(Dumbrell et al 2011)	Temperate grassland	454 sequencing of AMF SSU	11 samplings in 8 months	Root	AMF community composition differed between summer and winter; AMF beta diversity declined from Nov. to July.
(Montero Sommerfeld et al 2013)*	Grasslands in Chile	tRFLP	August, January	Root	AMF richness and composition significantly different between winter and summer
(Hazard et al 2014)	Pasture and arable field in Ireland	tRFLP	Six samplings in two years (Mar, June, Oct, Jan, Mar, Oct)	Root	AMF richness and composition changed by season
(Barnes et al 2016b)	Short rotation coppice willow plantation in UK	tRFLP	Four times a year (Oct, June, Aug, Oct)	Root	The spatial distance-decay pattern of AMF community changed by time
(Barnes et al 2016a)	*Miscanthus giganteus* plantation in UK	tRFLP	Four times a year (Oct, June, Aug, Oct)	Root	AMF richness and community composition changed over time
(Bouffaud et al 2017)	Four long-term observatories in Europe	454 sequencing of AMF ITS2	Spring, autumn	Soil	AMF community composition is weakly affected by season

(Husband et al 2002a)	Tropical forest (seedlings of two spp. in two sites)	Sanger sequencing of AMF SSU	Two years	Root	AMF community composition changed between years
(Taylor et al 2014)*	Boreal forest	Sanger sequencing of ITS	Two years	Soil	AMF community composition not changed by time, as reanalyzed by
(Husband et al 2002b)	Tropical forest (seedling)	Sanger sequencing of AMF SSU	Four samplings (3 month, 1, 2 and 5 yr)	Root	AMF community composition changed by time
(Helgason et al 1999)	Woodland in UK	RFLP	July vs Dec.	Root	AMF community composition show difference between seasons
(Kabir et al 1997)	Corn grown in Canada	Hyphae	Apr, Jun, Aug and Oct of two years	Root Soil	The levels of intra- and extraradical fungal colonization always increased from spring to silking and decreased thereafter.
(Kivlin and Hawkes 2016)	Monoculture stands of four tree species in Costa Rica	454 sequencing of fungal LSU	Dry and wet season, two years	Soil	AMF richness and PD affected by time
(Daniell et al 2001)	Arable fields around North Yorkshire, UK	Sanger sequencing of SSU	Nine sample times in 14 months	Root	AMF composition changed by time
(Bencherif et al 2016)	Algerian steppic area	Spore	Four seasons	Soil Root	Season affect AMF spore abundance and root colonization rate, but not diversity
(Herrmann et al 2016)	Tropical rubber tree plantation	454 sequencing of AMF SSU	Chronosequence (3, 6, 16 yr)	Root	AMF community composition differed between 3 and 16 yr trees in ordination constrained by soil variables, but not in unconstrained ordination
(Krüger et al 2017)	Temperate woodland recovered from brown-coal mining	454 sequencing of AMF LSU	Spring autumn, Chronosequence (12, 20, 30, 50 yr)	Root	AMF richness and community composition not affected by the chronosequences, but community variation increased along the chronosequences

(Yu et al 2017)	Semiarid grassland and woodland in China	Spore	Chronosequence (12 yr v.s. 30yr planted Caragana microphylla)	Soil	No significant differences in community composition and diversity of AM fungi were recorded at the dunes with different revegetation duration.
(Liu et al 2009)	Temperate tree plantation in China	DGGE	Three seasons Chronosequence (5, 13, 20, 42 yrs)	Root	AMF richness was not influenced by the chronosequences, but AMF community composition was affected by season
(Sheng et al 2017)	Temperate black locust plantations in China	454 sequencing of SSU	Chronosequence (11, 23, 35, 46 yr)	Root Soil	AMF community composition changed by plant age. AMF spore density increased with plant age. AMF richness not linearly related to plant age
(Hart et al 2014)	Tropical long-lived perennial breadfruit trees	454 sequencing of AMF SSU	Chronosequence (5-6, 20-21, 42-40 yr)	Root Soil	AMF richness increased with age in root, but not in soil; AMF community composition differ between young and old trees
(Guadarrama et al 2014)	Mexican seasonal dry forests	Spore	Chronosequence (<5 yr, 11-23 yr, >30 yr) Wet vs dry season	Soil	AMF diversity was affected by season and age
(García de León et al 2016b)	Temperate alvar grasslands	454 sequencing of AMF SSU	Chronosequence (young (20 yr), intermediate (50 yr), mature)	Soil Root	AMF community composition differed between young and mature grasslands, but not between intermediate and mature grasslands.
(Honnay et al 2017)	Grassland of Belgium	454 sequencing of SSU	Chronosequence (Forested, 8-11yr, 12-20yr, ancient)	Root Soil	AMF richness and community composition were affected by successional stages
(Roy et al 2017)	Recultivation after open-cast mining in Germany	Illumina sequencing of AMF LSU	Chronosequence (2013-2015, 2011-2012, 1964-2006)	Soil	AMF community composition differed among the three phases
(Johnson et al 1991)	Abandoned fields in Minnesota	Spore	Chronosequence (12 samples of 1-60 years)	Soil	AMF richness was not influenced by the chronosequence, but AMF Shannon's diversity index increased

(Kowalchuk et al 2002)	Dunes in Netherlands	DGGE	Chronosequence (Vigorous vs degenerating stand)	Root Soil	AMF diversity is lower in the later, degraded stages
(Oba et al 2004)	Recovery from volcanic deposits in Philippines	Spore	Chronosequence (Sites with sparse or dense vegetation)	Soil	AMF diversity and composition were not significantly differed between two sites
(Pezzani et al 2006)	Two-phase mosaics in Mexican Chihuahuan Desert	Spore	Chronosequence (Pioneer vs late-successional grasses)	Soil	Spore density was higher in late than in early successional stages
(Wu et al 2007)	Primary successional volcanic desert in Japan	Spore	Chronosequence (Different altitudes)	Root Soil	AMF spore abundance, richness of morphtypes increased with decreasing altitue
(Oehl et al 2011)	Retreat of Glacier in Alps	Trap culture	Chronosequence (1875–1900, 1940–1950, 1970–1980 and 1990–2000)	Soil	AMF diversity increased with succession
(Sikes et al 2012)	Sand dune in Michigan, USA	Sanger sequencing	Chronosequence (10- 35 yr, 235- 295 yr, 450-845 yr)	Soil	AMF isolated from early succession were more phylogenetically diverse relative to intermediate and late succession while late successional fungi consistently produced more soil hyphae and arbuscules.
(Gorzelak et al 2017)	Temperate rainforests of British Columbia	454 sequencing of LSU	Chronosequence Young, mature and old)	Root Soil	No differences in richness along the host chronosequence. AMF community composition was affected by age weakly. All host age classes harboured AMF communities that were overdispersed
(Bennett et al 2013)	Re-analysis of and		Chronosequence (Young, old) June, July, October		Succession affect connectance and H2, sampling time affect link/specie of plant-AMF symbiotic network

(Krüger et al 2015)	Dunes in Australia	454 sequencing of LSU	Chronosequence (1000 yr, 120 000 yr, > 2 000 000 yr soil)	Root<br>Soil	AMF richness peaked in the middle age. AMF community composition differed among the three stages
(Martínez-García et al 2015)	New Zealand	tRFLP, Sanger sequencing and 454 sequencing of SSU	Chronosequence (15 yr, 5000 yr, 12 000 yr, 60 000 – 120 000 yr)	Root	AMF community composition changed by successional stage
(Koziol and Bever 2016)	12 plant spp. forming a successional gradient				Mycorrhizal responsiveness change with plant successional status
(Senés-Guerrero and Schüßler 2016)	Potato<br>Bolivia, Ecuador and Peru	454 sequencing of LSU	emergence, flowering and senescence<br>105 samples = 3 sites * 3 stages * 3 replicates * 4 altitudes	Root	a surprisingly conserved AMF core-species community structure in Andean potatoes, regardless of different plant stages and environmental factors

*Re-analyzed by Bahram et al (2015); **Also re-analyzed by this study in Fig. 2. SSU: small subunit; LSU, large subunit; ITS: internal transcribed spacer; DGGE: Denaturing Gradient Gel Electrophoresis; tRFLP, terminal restriction fragment length polymorphism.

Table S2 The forward and reverse primers that we constructed to be used for Illumina Miseq PE300 in this study. Note we constructed 24 forward and 24 reverse primers that, via a dual-indexing approach, enable us to sequence up to 576 samples in a Miseq PE 300 lane.

**Primer Name**	**LINKER**	**BARCODE**	**PAD**	**SPACER**	**PRIMER**	**COMPLETE**
**5.8SFun_S_24_01**	**AATGATACGGCGACCACCGAGATCTACAC**	**CCTAAACTACGG**	**TCTTTCCCTACACGACGCTCTTCCGATCT**		**AACTTTYRRCAAYGGATCWCT**	**AATGATACGGCGACCACCGAGATCTACACCCTAAACTACGGTCTTTCCCTACACGACGCTCTTCCGATCTAACTTTYRRCAAYGGATCWCT**
**5.8SFun_S_24_02**	**AATGATACGGCGACCACCGAGATCTACAC**	**GTGGTATGGGAG**	**TCTTTCCCTACACGACGCTCTTCCGATCT**	**T**	**AACTTTYRRCAAYGGATCWCT**	**AATGATACGGCGACCACCGAGATCTACACGTGGTATGGGAGTCTTTCCCTACACGACGCTCTTCCGATCTTAACTTTYRRCAAYGGATCWCT**
**5.8SFun_S_24_03**	**AATGATACGGCGACCACCGAGATCTACAC**	**TGTTGCGTTTCT**	**TCTTTCCCTACACGACGCTCTTCCGATCT**	**GT**	**AACTTTYRRCAAYGGATCWCT**	**AATGATACGGCGACCACCGAGATCTACACTGTTGCGTTTCTTCTTTCCCTACACGACGCTCTTCCGATCTGTAACTTTYRRCAAYGGATCWCT**
**5.8SFun_S_24_04**	**AATGATACGGCGACCACCGAGATCTACAC**	**ACAGCCACCCAT**	**TCTTTCCCTACACGACGCTCTTCCGATCT**	**CGA**	**AACTTTYRRCAAYGGATCWCT**	**AATGATACGGCGACCACCGAGATCTACACACAGCCACCCATTCTTTCCCTACACGACGCTCTTCCGATCTCGAAACTTTYRRCAAYGGATCWCT**
**5.8SFun_S_24_05**	**AATGATACGGCGACCACCGAGATCTACAC**	**GTTACGTGGTTG**	**TCTTTCCCTACACGACGCTCTTCCGATCT**	**ATGA**	**AACTTTYRRCAAYGGATCWCT**	**AATGATACGGCGACCACCGAGATCTACACGTTACGTGGTTGTCTTTCCCTACACGACGCTCTTCCGATCTATGAAACTTTYRRCAAYGGATCWCT**
**5.8SFun_S_24_06**	**AATGATACGGCGACCACCGAGATCTACAC**	**TACCGGCTTGCA**	**TCTTTCCCTACACGACGCTCTTCCGATCT**	**TGCGA**	**AACTTTYRRCAAYGGATCWCT**	**AATGATACGGCGACCACCGAGATCTACACTACCGGCTTGCATCTTTCCCTACACGACGCTCTTCCGATCTTGCGAAACTTTYRRCAAYGGATCWCT**
**5.8SFun_S_24_07**	**AATGATACGGCGACCACCGAGATCTACAC**	**TGCAGATCCAAC**	**TCTTTCCCTACACGACGCTCTTCCGATCT**	**GAGTGG**	**AACTTTYRRCAAYGGATCWCT**	**AATGATACGGCGACCACCGAGATCTACACTGCAGATCCAACTCTTTCCCTACACGACGCTCTTCCGATCTGAGTGGAACTTTYRRCAAYGGATCWCT**
**5.8SFun_S_24_08**	**AATGATACGGCGACCACCGAGATCTACAC**	**TTAACTGGAAGC**	**TCTTTCCCTACACGACGCTCTTCCGATCT**	**CCTGGAG**	**AACTTTYRRCAAYGGATCWCT**	**AATGATACGGCGACCACCGAGATCTACACTTAACTGGAAGCTCTTTCCCTACACGACGCTCTTCCGATCTCCTGGAGAACTTTYRRCAAYGGATCWCT**

5.8SFun_S_24_09	AATGATACGGCGACCACCGAGATCTACAC	TACCGCCTCGGA	TCTTTCCCTACACGACGCTCTTCCGATCT		AACTTTYRRCAAYGGATCWCT	AATGATACGGCGACCACCGAGATCTACACTACCGCCTCGGATCTTTCCCTACACGACGCTCTTCCGATCTAACTTTYRRCAAYGGATCWCT
5.8SFun_S_24_10	AATGATACGGCGACCACCGAGATCTACAC	ACTTTAAGGGTG	TCTTTCCCTACACGACGCTCTTCCGATCT	T	AACTTTYRRCAAYGGATCWCT	AATGATACGGCGACCACCGAGATCTACACACTTTAAGGGTGTCTTTCCCTACACGACGCTCTTCCGATCTTAACTTTYRRCAAYGGATCWCT
5.8SFun_S_24_11	AATGATACGGCGACCACCGAGATCTACAC	CCATCACATAGG	TCTTTCCCTACACGACGCTCTTCCGATCT	GT	AACTTTYRRCAAYGGATCWCT	AATGATACGGCGACCACCGAGATCTACACCCATCACATAGGTCTTTCCCTACACGACGCTCTTCCGATCTGTAACTTTYRRCAAYGGATCWCT
5.8SFun_S_24_12	AATGATACGGCGACCACCGAGATCTACAC	GAGCAACATCCT	TCTTTCCCTACACGACGCTCTTCCGATCT	CGA	AACTTTYRRCAAYGGATCWCT	AATGATACGGCGACCACCGAGATCTACACGAGCAACATCCTTCTTTCCCTACACGACGCTCTTCCGATCTCGAAACTTTYRRCAAYGGATCWCT
5.8SFun_S_24_13	AATGATACGGCGACCACCGAGATCTACAC	ATGTCCGACCAA	TCTTTCCCTACACGACGCTCTTCCGATCT	ATGA	AACTTTYRRCAAYGGATCWCT	AATGATACGGCGACCACCGAGATCTACACATGTCCGACCAATCTTTCCCTACACGACGCTCTTCCGATCTATGAAACTTTYRRCAAYGGATCWCT
5.8SFun_S_24_14	AATGATACGGCGACCACCGAGATCTACAC	TGTCTCGCAAGC	TCTTTCCCTACACGACGCTCTTCCGATCT	TGCGA	AACTTTYRRCAAYGGATCWCT	AATGATACGGCGACCACCGAGATCTACACTGTCTCGCAAGCTCTTTCCCTACACGACGCTCTTCCGATCTTGCGAAACTTTYRRCAAYGGATCWCT
5.8SFun_S_24_15	AATGATACGGCGACCACCGAGATCTACAC	CGCGGTTACTAA	TCTTTCCCTACACGACGCTCTTCCGATCT	GAGTGG	AACTTTYRRCAAYGGATCWCT	AATGATACGGCGACCACCGAGATCTACACCGCGGTTACTAATCTTTCCCTACACGACGCTCTTCCGATCTGAGTGGAACTTTYRRCAAYGGATCWCT
5.8SFun_S_24_16	AATGATACGGCGACCACCGAGATCTACAC	GAGACTATATGC	TCTTTCCCTACACGACGCTCTTCCGATCT	CCTGGAG	AACTTTYRRCAAYGGATCWCT	AATGATACGGCGACCACCGAGATCTACACGAGACTATATGCTCTTTCCCTACACGACGCTCTTCCGATCTCCTGGAGAACTTTYRRCAAYGGATCWCT
5.8SFun_S_24_17	AATGATACGGCGACCACCGAGATCTACAC	AGGTACGCAATT	TCTTTCCCTACACGACGCTCTTCCGATCT		AACTTTYRRCAAYGGATCWCT	AATGATACGGCGACCACCGAGATCTACACAGGTACGCAATTTCTTTCCCTACACGACGCTCTTCCGATCTAACTTTYRRCAAYGGATCWCT
5.8SFun_S_24_18	AATGATACGGCGACCACCGAGATCTACAC	GAGGAGTAAAGC	TCTTTCCCTACACGACGCTCTTCCGATCT	T	AACTTTYRRCAAYGGATCWCT	AATGATACGGCGACCACCGAGATCTACACGAGGAGTAAAGCTCTTTCCCTACACGACGCTCTTCCGATCTTAACTTTYRRCAAYGGATCWCT

**5.8SFun_S_24_19**	**AATGATACGGCGACCACCGAGATCTACAC**	**CGTAAGATGCCT**	**TCTTTCCCTACACGACGCTCTTCCGATCT**	**GT**	**AACTTTYRRCAAYGGATCWCT**	**AATGATACGGCGACCACCGAGATCTACACCGTAAGATGCCTTCTTTCCCTACACGACGCTCTTCCGATCTGTAACTTTYRRCAAYGGATCWCT**
**5.8SFun_S_24_20**	**AATGATACGGCGACCACCGAGATCTACAC**	**ATCTAGTGGCAA**	**TCTTTCCCTACACGACGCTCTTCCGATCT**	**CGA**	**AACTTTYRRCAAYGGATCWCT**	**AATGATACGGCGACCACCGAGATCTACACATCTAGTGGCAATCTTTCCCTACACGACGCTCTTCCGATCTCGAAACTTTYRRCAAYGGATCWCT**
**5.8SFun_S_24_21**	**AATGATACGGCGACCACCGAGATCTACAC**	**CCAGGGACTTCT**	**TCTTTCCCTACACGACGCTCTTCCGATCT**	**ATGA**	**AACTTTYRRCAAYGGATCWCT**	**AATGATACGGCGACCACCGAGATCTACACCCAGGGACTTCTTCTTTCCCTACACGACGCTCTTCCGATCTATGAAACTTTYRRCAAYGGATCWCT**
**5.8SFun_S_24_22**	**AATGATACGGCGACCACCGAGATCTACAC**	**CACCTTACCTTA**	**TCTTTCCCTACACGACGCTCTTCCGATCT**	**TGCGA**	**AACTTTYRRCAAYGGATCWCT**	**AATGATACGGCGACCACCGAGATCTACACCACCTTACCTTATCTTTCCCTACACGACGCTCTTCCGATCTTGCGAAACTTTYRRCAAYGGATCWCT**
**5.8SFun_S_24_23**	**AATGATACGGCGACCACCGAGATCTACAC**	**ATAGTTAGGGCT**	**TCTTTCCCTACACGACGCTCTTCCGATCT**	**GAGTGG**	**AACTTTYRRCAAYGGATCWCT**	**AATGATACGGCGACCACCGAGATCTACACATAGTTAGGGCTTCTTTCCCTACACGACGCTCTTCCGATCTGAGTGGAACTTTYRRCAAYGGATCWCT**
**5.8SFun_S_24_24**	**AATGATACGGCGACCACCGAGATCTACAC**	**GCACTTCATTTC**	**TCTTTCCCTACACGACGCTCTTCCGATCT**	**CCTGGAG**	**AACTTTYRRCAAYGGATCWCT**	**AATGATACGGCGACCACCGAGATCTACACGCACTTCATTTCTCTTTCCCTACACGACGCTCTTCCGATCTCCTGGAGAACTTTYRRCAAYGGATCWCT**
**ITS4Fun_S_24_01**	**CAAGCAGAAGACGGCATACGAGAT**	**CCTAAACTACGG**	**GTGACTGGAGTTCAGACGTGTGCTCTTCCGATCT**		**AGCCTCCGCTTATTGATATGCTTAART**	**CAAGCAGAAGACGGCATACGAGATCCTAAACTACGGGTGACTGGAGTTCAGACGTGTGCTCTTCCGATCTAGCCTCCGCTTATTGATATGCTTAART**
**ITS4Fun_S_24_02**	**CAAGCAGAAGACGGCATACGAGAT**	**GTGGTATGGGAG**	**GTGACTGGAGTTCAGACGTGTGCTCTTCCGATCT**	**G**	**AGCCTCCGCTTATTGATATGCTTAART**	**CAAGCAGAAGACGGCATACGAGATGTGGTATGGGAGGTGACTGGAGTTCAGACGTGTGCTCTTCCGATCTGAGCCTCCGCTTATTGATATGCTTAART**
**ITS4Fun_S_24_03**	**CAAGCAGAAGACGGCATACGAGAT**	**TGTTGCGTTTCT**	**GTGACTGGAGTTCAGACGTGTGCTCTTCCGATCT**	**TC**	**AGCCTCCGCTTATTGATATGCTTAART**	**CAAGCAGAAGACGGCATACGAGATTGTTGCGTTTCTGTGACTGGAGTTCAGACGTGTGCTCTTCCGATCTTCAGCCTCCGCTTATTGATATGCTTAART**
**ITS4Fun_S_24_04**	**CAAGCAGAAGACGGCATACGAGAT**	**ACAGCCACCCAT**	**GTGACTGGAGTTCAGACGTGTGCTCTTCCGATCT**	**CTA**	**AGCCTCCGCTTATTGATATGCTTAART**	**CAAGCAGAAGACGGCATACGAGATACAGCCACCCATGTGACTGGAGTTCAGACGTGTGCTCTTCCGATCTCTAAGCCTCCGCTTATTGATATGCTTAART**

ITS4Fun_S_24_05	CAAGCAGAAGACGGCATACGAGAT	GTTACGTGGTTG	GTGACTGGAGTTCAGACGTGTGCTCTTCCGATCT	GATA	AGCCTCCGCTTATTGATATGCTTAART	CAAGCAGAAGACGGCATACGAGATGTTACGTGGTTGGTGACTGGAGTTCAGACGTGTGCTCTTCCGATCTGATAAGCCTCCGCTTATTGATATGCTTAART
ITS4Fun_S_24_06	CAAGCAGAAGACGGCATACGAGAT	TACCGGCTTGCA	GTGACTGGAGTTCAGACGTGTGCTCTTCCGATCT	ACTCA	AGCCTCCGCTTATTGATATGCTTAART	CAAGCAGAAGACGGCATACGAGATTACCGGCTTGCAGTGACTGGAGTTCAGACGTGTGCTCTTCCGATCTACTCAAGCCTCCGCTTATTGATATGCTTAART
ITS4Fun_S_24_07	CAAGCAGAAGACGGCATACGAGAT	CACCTTACCTTA	GTGACTGGAGTTCAGACGTGTGCTCTTCCGATCT	TTCTCT	AGCCTCCGCTTATTGATATGCTTAART	CAAGCAGAAGACGGCATACGAGATCACCTTACCTTAGTGACTGGAGTTCAGACGTGTGCTCTTCCGATCTTTCTCTAGCCTCCGCTTATTGATATGCTTAART
ITS4Fun_S_24_08	CAAGCAGAAGACGGCATACGAGAT	TTAACTGGAAGC	GTGACTGGAGTTCAGACGTGTGCTCTTCCGATCT	CACTTCT	AGCCTCCGCTTATTGATATGCTTAART	CAAGCAGAAGACGGCATACGAGATTTAACTGGAAGCGTGACTGGAGTTCAGACGTGTGCTCTTCCGATCTCACTTCTAGCCTCCGCTTATTGATATGCTTAART
ITS4Fun_S_24_09	CAAGCAGAAGACGGCATACGAGAT	TACCGCCTCGGA	GTGACTGGAGTTCAGACGTGTGCTCTTCCGATCT		AGCCTCCGCTTATTGATATGCTTAART	CAAGCAGAAGACGGCATACGAGATTACCGCCTCGGAGTGACTGGAGTTCAGACGTGTGCTCTTCCGATCTAGCCTCCGCTTATTGATATGCTTAART
ITS4Fun_S_24_10	CAAGCAGAAGACGGCATACGAGAT	ACTTTAAGGGTG	GTGACTGGAGTTCAGACGTGTGCTCTTCCGATCT	G	AGCCTCCGCTTATTGATATGCTTAART	CAAGCAGAAGACGGCATACGAGATACTTTAAGGGTGGTGACTGGAGTTCAGACGTGTGCTCTTCCGATCTGAGCCTCCGCTTATTGATATGCTTAART
ITS4Fun_S_24_11	CAAGCAGAAGACGGCATACGAGAT	CCATCACATAGG	GTGACTGGAGTTCAGACGTGTGCTCTTCCGATCT	TC	AGCCTCCGCTTATTGATATGCTTAART	CAAGCAGAAGACGGCATACGAGATCCATCACATAGGGTGACTGGAGTTCAGACGTGTGCTCTTCCGATCTTCAGCCTCCGCTTATTGATATGCTTAART
ITS4Fun_S_24_12	CAAGCAGAAGACGGCATACGAGAT	GAGCAACATCCT	GTGACTGGAGTTCAGACGTGTGCTCTTCCGATCT	CTA	AGCCTCCGCTTATTGATATGCTTAART	CAAGCAGAAGACGGCATACGAGATGAGCAACATCCTGTGACTGGAGTTCAGACGTGTGCTCTTCCGATCTCTAAGCCTCCGCTTATTGATATGCTTAART
ITS4Fun_S_24_13	CAAGCAGAAGACGGCATACGAGAT	ATGTCCGACCAA	GTGACTGGAGTTCAGACGTGTGCTCTTCCGATCT	GATA	AGCCTCCGCTTATTGATATGCTTAART	CAAGCAGAAGACGGCATACGAGATATGTCCGACCAAGTGACTGGAGTTCAGACGTGTGCTCTTCCGATCTGATAAGCCTCCGCTTATTGATATGCTTAART
ITS4Fun_S_24_14	CAAGCAGAAGACGGCATACGAGAT	TGTCTCGCAAGC	GTGACTGGAGTTCAGACGTGTGCTCTTCCGATCT	ACTCA	AGCCTCCGCTTATTGATATGCTTAART	CAAGCAGAAGACGGCATACGAGATTGTCTCGCAAGCGTGACTGGAGTTCAGACGTGTGCTCTTCCGATCTACTCAAGCCTCCGCTTATTGATATGCTTAART

ITS4Fun_S_24_15	CAAGCAGAAGACGGCATACGAGAT	CGCGGTTACTAA	GTGACTGGAGTTCAGACGTGTGCTCTTCCGATCT	TTCTCT	AGCCTCCGCTTATTGATATGCTTAART	CAAGCAGAAGACGGCATACGAGATCGCGGTTACTAAGTGACTGGAGTTCAGACGTGTGCTCTTCCGATCTTTCTCTAGCCTCCGCTTATTGATATGCTTAART
ITS4Fun_S_24_16	CAAGCAGAAGACGGCATACGAGAT	GAGACTATATGC	GTGACTGGAGTTCAGACGTGTGCTCTTCCGATCT	CACTTCT	AGCCTCCGCTTATTGATATGCTTAART	CAAGCAGAAGACGGCATACGAGATGAGACTATATGCGTGACTGGAGTTCAGACGTGTGCTCTTCCGATCTCACTTCTAGCCTCCGCTTATTGATATGCTTAART
ITS4Fun_S_24_17	CAAGCAGAAGACGGCATACGAGAT	AGGTACGCAATT	GTGACTGGAGTTCAGACGTGTGCTCTTCCGATCT		AGCCTCCGCTTATTGATATGCTTAART	CAAGCAGAAGACGGCATACGAGATAGGTACGCAATTGTGACTGGAGTTCAGACGTGTGCTCTTCCGATCTAGCCTCCGCTTATTGATATGCTTAART
ITS4Fun_S_24_18	CAAGCAGAAGACGGCATACGAGAT	GAGGAGTAAAGC	GTGACTGGAGTTCAGACGTGTGCTCTTCCGATCT	G	AGCCTCCGCTTATTGATATGCTTAART	CAAGCAGAAGACGGCATACGAGATGAGGAGTAAAGCGTGACTGGAGTTCAGACGTGTGCTCTTCCGATCTGAGCCTCCGCTTATTGATATGCTTAART
ITS4Fun_S_24_19	CAAGCAGAAGACGGCATACGAGAT	CGTAAGATGCCT	GTGACTGGAGTTCAGACGTGTGCTCTTCCGATCT	TC	AGCCTCCGCTTATTGATATGCTTAART	CAAGCAGAAGACGGCATACGAGATCGTAAGATGCCTGTGACTGGAGTTCAGACGTGTGCTCTTCCGATCTTCAGCCTCCGCTTATTGATATGCTTAART
ITS4Fun_S_24_20	CAAGCAGAAGACGGCATACGAGAT	ATCTAGTGGCAA	GTGACTGGAGTTCAGACGTGTGCTCTTCCGATCT	CTA	AGCCTCCGCTTATTGATATGCTTAART	CAAGCAGAAGACGGCATACGAGATATCTAGTGGCAAGTGACTGGAGTTCAGACGTGTGCTCTTCCGATCTCTAAGCCTCCGCTTATTGATATGCTTAART
ITS4Fun_S_24_21	CAAGCAGAAGACGGCATACGAGAT	CCAGGGACTTCT	GTGACTGGAGTTCAGACGTGTGCTCTTCCGATCT	GATA	AGCCTCCGCTTATTGATATGCTTAART	CAAGCAGAAGACGGCATACGAGATCCAGGGACTTCTGTGACTGGAGTTCAGACGTGTGCTCTTCCGATCTGATAAGCCTCCGCTTATTGATATGCTTAART
ITS4Fun_S_24_22	CAAGCAGAAGACGGCATACGAGAT	TGCAGATCCAAC	GTGACTGGAGTTCAGACGTGTGCTCTTCCGATCT	ACTCA	AGCCTCCGCTTATTGATATGCTTAART	CAAGCAGAAGACGGCATACGAGATTGCAGATCCAACGTGACTGGAGTTCAGACGTGTGCTCTTCCGATCTACTCAAGCCTCCGCTTATTGATATGCTTAART
ITS4Fun_S_24_23	CAAGCAGAAGACGGCATACGAGAT	ATAGTTAGGGCT	GTGACTGGAGTTCAGACGTGTGCTCTTCCGATCT	TTCTCT	AGCCTCCGCTTATTGATATGCTTAART	CAAGCAGAAGACGGCATACGAGATATAGTTAGGGCTGTGACTGGAGTTCAGACGTGTGCTCTTCCGATCTTTCTCTAGCCTCCGCTTATTGATATGCTTAART
ITS4Fun_S_24_24	CAAGCAGAAGACGGCATACGAGAT	GCACTTCATTTC	GTGACTGGAGTTCAGACGTGTGCTCTTCCGATCT	CACTTCT	AGCCTCCGCTTATTGATATGCTTAART	CAAGCAGAAGACGGCATACGAGATGCACTTCATTTCGTGACTGGAGTTCAGACGTGTGCTCTTCCGATCTCACTTCTAGCCTCCGCTTATTGATATGCTTAART

Table S3 Molecular identification of arbuscular mycorrhizal fungi in this study

OTU	Coverage	E-value	Similarity	Best NCBI hit	
OTU51_Rhizophagus	99%	2.00E-159	93%	JN195441.1	Uncultured_Glomus
OTU70_Claroideoglomus	99%	0	99%	JF439206.1	Glomus_hoi
OTU166_Rhizophagus	98%	0	98%	JN936299.1	Rhizophagus_sp.
OTU150_Rhizophagus	98%	0	97%	JN936299.1	Rhizophagus_sp.
OTU253_Funneliformis	98%	0	99%	AJ919274.1	Glomus_mosseae
OTU118_Rhizophagus*	98%	4.00E-180	98%	KM041773.1	Uncultured_Rhizophagus
OTU161_Rhizophagus*	98%	1.00E-173	97%	KM041753.1	Uncultured_Rhizophagus
OTU132_Rhizophagus*	98%	0	98%	JX999965.1	Glomeromycota_sp.
OTU133_Glomus	98%	2.00E-172	96%	KM208159.1	Uncultured_Glomeromycota
OTU206_Glomus	99%	2.00E-178	97%	GQ388297.1	Uncultured_Glomus
OTU213_Rhizophagus	98%	0	98%	JX999971.1	Glomeromycota_sp.
OTU935_Rhizophagus	98%	1.00E-156	92%	JN195441.1	Uncultured_Glomus
OTU126_Glomus	98%	5.00E-173	97%	KM208171.1	Uncultured_Glomeromycota
OTU251_Rhizophagus	98%	0	98%	GQ205073.1	Glomus_custos
OTU558_Funneliformis	99%	0	98%	HG425925.1	Uncultured_Funneliformis
OTU323_Glomus	98%	1.00E-174	97%	KM208159.1	Uncultured_Glomeromycota
OTU224_Glomus	99%	7.00E-178	98%	GQ388314.1	Uncultured_Glomus
OTU229_Glomus	98%	2.00E-179	98%	KM208159.1	Uncultured_Glomeromycota
OTU355_Funneliformis	99%	0	98%	HF970318.1	Uncultured_Funneliformis
OTU347_Rhizophagus	98%	0	98%	KJ701452.1	Uncultured_Glomus
OTU476_Glomus	99%	4.00E-180	97%	HG425896.1	Uncultured_Glomeraceae
OTU277_Glomus	99%	7.00E-178	98%	GQ388297.1	Uncultured_Glomus
OTU565_Funneliformis	99%	0	98%	U49264.1	Glomus_moessae
OTU491_Claroideoglomus	99%	0	99%	KY927389.1	Claroideoglomus_etunicatum
OTU400_Glomus	99%	7.00E-178	97%	JX276899.1	Uncultured_Glomeromycota
OTU333_Glomus	99%	0	98%	FR693682.1	Uncultured_Glomus

OTU435_Glomus	99%	0	98%	HG425982.1	Uncultured_Glomeraceae
OTU534_Glomus	98%	0	99%	KF836947.1	Glomus_sp.
OTU944_Rhizophagus	99%	1.00E-154	91%	JN195441.1	Uncultured_Glomus
OTU536_Claroideoglomus	98%	0	97%	JQ218217.1	Uncultured_Glomus
OTU989_Funneliformis	99%	0	98%	EF989113.1	Funneliformis_mosseae
OTU498_Glomus	99%	3.00E-176	98%	JX096590.1	Uncultured_Glomeromycota
OTU579_Glomus	99%	6.00E-166	96%	JX096590.1	Uncultured_Glomeromycota
OTU987_Paraglomus	99%	7.00E-159	88%	AB520480.1	Uncultured_fungus
OTU734_Rhizophagus	97%	0	98%	AF185651.1	Glomus_intraradices
OTU715_Glomus	99%	1.00E-173	97%	JX096590.1	Uncultured_Glomeromycota
OTU574_Glomus	98%	1.00E-174	97%	JX096590.1	Uncultured_Glomeromycota
OTU659_Glomus	98%	5.00E-179	98%	JX096614.1	Uncultured_Glomeromycota
OTU1068_Glomus	98%	1.00E-173	97%	HM162343.1	Uncultured_Glomeromycota
OTU564_Glomus	99%	2.00E-160	95%	GU059545.1	Glomus_indicum
OTU735_Claroideoglomus	99%	0	96%	AF004682.1	Glomus_etunicatum
OTU597_Paraglomus	98%	6.00E-166	91%	AB520480.1	Uncultured_fungus
OTU998_Glomus	99%	8.00E-177	97%	GQ388297.1	Uncultured_Glomus
OTU1184_Rhizophagus	98%	0	99%	AF185650.1	Glomus_intraradices
OTU1065_Claroideoglomus	99%	0	96%	KP191488.1	Claroideoglomus_drummondii
OTU936_Claroideoglomus	100%	0	99%	JN685281.1	Uncultured_Glomeromycota
OTU1197_Claroideoglomus	99%	0	97%	KP191486.1	Claroideoglomus_drummondii
OTU1274_Paraglomus	98%	9.00E-151	85%	AB520480.1	Uncultured_fungus
OTU969_Claroideoglomus	98%	0	97%	JX096582.1	Uncultured_Glomeromycota
OTU1013_Glomeraceae	98%	4.00E-180	96%	JN195694.1	Uncultured_Glomus
OTU975_Paraglomus	99%	0	99%	KF849701.1	Uncultured_Paraglomus
OTU945_Glomus	99%	0	99%	KF849595.1	Uncultured_Glomus

*Note: These three OTUs are combined to avoid the potential more than one rDNA repeat in a single species. However, the ecological results and conclusion are not affected (Fig. S10-S15). Representative sequences of AMF OTUs were deposited in GenBank with the accession codes: MG008508 - MG008559.

Table S4 Arbuscular mycorrhizal fungal (AMF) operational taxonomic units (OTUs) bias occurred in the first week (TP01) and the last week (TP17), as detected by indicator species analysis. Note the steep decline of two initially dominant species and the rise of 13 initially rare *Rhizophagus* and *Glomus* species

AMF OTUs	Preferred	Indicator value	*P*
OTU51_*Rhizophagus*	TP01	0.894	0.001
OTU70_*Claroideoglomus*	TP01	0.809	0.001
OTU166_*Rhizophagus*	TP17	0.855	0.001
OTU118.161.132_*Rhizophagus*	TP17	0.811	0.001
OTU150_*Rhizophagus*	TP17	0.797	0.001
OTU213_*Rhizophagus*	TP17	0.75	0.001
OTU251_*Rhizophagus*	TP17	0.667	0.001
OTU126_*Glomus*	TP17	0.816	0.001
OTU133_*Glomus*	TP17	0.788	0.001
OTU229_*Glomus*	TP17	0.762	0.001
OTU323_*Glomus*	TP17	0.711	0.001
OTU476_*Glomus*	TP17	0.474	0.007
OTU333_*Glomus*	TP17	0.415	0.01
OTU534_*Glomus*	TP17	0.318	0.05
OTU400_*Glomus*	TP17	0.311	0.035

Table S5 Arbuscular mycorrhizal fungal (AMF) operational taxonomic units (OTUs) bias occurred in the root, rhizosphere and soil, as detected by indicator species analysis. Note a number of *Rhizophagus* were more common and abundant in root, whereas *Funneliformis*, *Claroideoglomus*, *Paraglomus* and *Glomus* were more common and abundant in rhizosphere and soil

AMF OTUs	Indicator	Indicator value	*P*
OTU118.161.132_*Rhizophagus*	Root	0.399	0.001
OTU166_*Rhizophagus*	Root	0.391	0.001
OTU213_*Rhizophagus*	Root	0.37	0.001
OTU150_*Rhizophagus*	Root	0.363	0.002
OTU251_*Rhizophagus*	Root	0.236	0.009
OTU734_*Rhizophagus*	Root	0.054	0.011
OTU70_*Claroideoglomus*	Rhizosphere	0.419	0.001
OTU206_*Glomus*	Soil	0.566	0.001
OTU224_*Glomus*	Soil	0.529	0.001
OTU277_*Glomus*	Soil	0.434	0.001
OTU498_*Glomus*	Soil	0.118	0.001
OTU659_*Glomus*	Soil	0.104	0.001
OTU998_*Glomus*	Soil	0.104	0.001
OTU579_*Glomus*	Soil	0.1	0.001
OTU574_*Glomus*	Soil	0.089	0.001
OTU564_*Glomus*	Soil	0.085	0.001
OTU1068_*Glomus*	Soil	0.068	0.001
OTU715_*Glomus*	Soil	0.062	0.005
OTU253_*Funneliformis*	Soil	0.52	0.001
OTU558_*Funneliformis*	Soil	0.277	0.001
OTU355_*Funneliformis*	Soil	0.261	0.001
OTU565_*Funneliformis*	Soil	0.222	0.001
OTU989_*Funneliformis*	Soil	0.103	0.006
OTU491_*Claroideoglomus*	Soil	0.167	0.001
OTU536_*Claroideoglomus*	Soil	0.088	0.007
OTU1065_*Claroideoglomus*	Soil	0.051	0.005
OTU987_*Paraglomus*	Soil	0.144	0.001
OTU597_*Paraglomus*	Soil	0.047	0.021

Table S6 A list of studies demonstrating the nestedness of arbuscular mycorrhizal fungal (AMF) community. Note nestedness is prevailing in AMF community, but not seen in some other studies

Study	Ecosystem	AMF recognition	Conclusion
(Kawahara et al 2016)	Six locations along a pH gradient in Japan	Trap culture, Sanger sequencing of LSU	AMF communities in lower pH soils were subsets of (nested in) those in higher pH soil
(van Geel et al 2015)	Apple trees in 24 orchards in Belgium	454 sequencing of AMF SSU	Degree of nestedness of the AMF communities was related to plant-available P and N content of the soil, pointing to a progressive loss of AMF taxa with increasing fertilization.
(Vályi et al 2015)	Land use intensity gradient in Germany	454 sequencing of AMF SSU	Communities in medium and low land-use sites were subsets of high land-use communities
(Camenzind et al 2014)	Tropical montane forest in Ecuador	454 sequencing of AMF LSU	AMF community is highly nested
(Verbruggen et al 2012)	40 agricultural soils in the Netherlands	tRFLP of LSU	Communities from species-poor fields were found to be subsets of those in richer fields
(Chen et al 2017a)	Subtropical forest in China	454 sequencing of AMF SSU	Plant-AMF symbiotic network is highly nested
(Chagnon et al 2012)	Reanalysis of study in hemiboreal forest	454 sequencing of AMF SSU	Plant-AMF symbiotic network is highly nested
(Montesinos-Navarro et al 2012)	Semiarid valley	Sanger sequencing of AMF ITS	Plant-AMF symbiotic network is highly nested
(Toju et al 2014)	Temperate forest	Sequencing of fungal ITS	No significant nestedness in plant-AMF symbiotic network
(Van Geel et al 2017a)	European grasslands	454 sequencing of AMF SSU	Plant-AMF symbiotic network is highly nested
(Van Geel et al 2017b)	European vineyards	454 sequencing of AMF SSU	AMF community is highly nested

SSU: small subunit; LSU, large subunit; ITS: internal transcribed spacer; tRFLP, terminal restriction fragment length polymorphism.

Table S7 List of studies investigating the phylogenetic relatedness of arbuscular mycorrhizal fungal (AMF) community. Note phylogenetic underdispersion, overdisperion and stochastic are all seen, with the underdisperion most prevalent

Study	Ecosystem	AMF recognition	Conclusion
(Bainard et al 2014)	Farmed pea, lentil and wheat in temperate semiarid prairie	454 sequencing of AMF SSU	AMF communities in general are phylogenetically underdispered, with exception of the first soil and second root samplings.
(Horn et al 2014)	Temperate grassland in Germany	454 sequencing of AMF LSU	AMF communities are phylogenetically underdispersed
(Chen et al 2017b)	Temperate semiarid steppe in China	454 sequencing of AMF SSU	AMF communities are phylogenetically underdispered, regardless of precipitation and nitrogen
(Liu et al 2015a)	Alpine meadow in China	Sanger sequencing of SSU	AMF shifted from phylogenetic underdispersion to overdisersion with increasing nitrogen fertilization
(García de León et al 2016a)	Abandoned quarry in Estonia	454 sequencing of AMF SSU	Phylogenetic community composition of AMF was more clustered than global, and European taxon pools
(Davison et al 2016)	Re-analysis of global dataset of	454 sequencing of AMF SSU	Coexisting fungi were more phylogenetically clustered than the random communities defined by a variety of null models.
(Maherali and Klironomos 2012)	Meadow (50 x 50 m^2 with 2601 samples) in Canada	Spores	AMF communities are generally phylogenetically overdispersed, but a subset of AMF communities are phylogenetically underdispersed
(Maherali and Klironomos 2007)	Greenhouse controlling initial AMF phylogenetic diversity	tRFLP	Initial phylogenetic overdispersion of AMF community result in high richness
(Roger et al 2013)	Pot cultures inoculated with *Rhizophafus irregularis* isolate combinations with different phylogenetic relatedness	qPCR	When fungi were closely related, they were able to coexist in almost equal proportions

(Liu et al 2015b)	Alpine meadow in China	Sanger sequencing of SSU	AMF communities were phylogenetically clustered and random in unfertilized and fertilized plots, respectively.
(Mueller and Bohannan 2015)	Grassland in California, USA	Sanger sequencing of SSU	AMF communities were generally phylogenetically underdispered, but not significant in the nitrogen treatment
(Egan et al 2017)	Subalpine grassland, treeline and alpine tundra along an elevational gradient in Montana, USA	454 sequencing of AMF SSU	AMF communities being phylogenetically clustered at all elevations sampled
(Saks et al 2013)	Mature mixed boreonemoral forest in Estonia	454 sequencing of AMF SSU	AMF communities being frequently phylogenetically clustered compared with local and global taxon pools
(Kivlin et al 2011)	Meta-analysis of 111 published studies	14,961 public DNA sequences	AMF communities being phylogenetically clustered in the majority of sites, and only two sites had communities that were phylogenetically dispersed
(Shi et al 2017)	Alpine meadow in China	454 sequencing of AMF SSU	AMF community was phylogenetically clustered under warming, but not in control, or plots experienced clip

SSU: small subunit; LSU, large subunit; RFLP: restriction fragment length polymorphism.

Table S8 List of studies comparing arbuscular mycorrhizal fungal (AMF) communities between root and soil. Note different root and soil AMF are often seen, but not in some studies

Study	Ecosystem	AMF recognition	Conclusion
(Hempel et al 2007)	Farmed meadow in Germany	Sanger sequencing of ITS	Root is abundant in *Rhizophagus* (*Glomus* group Ab); soil is abundant in Paraglomeraceae and Archaeosporaceae
(Bainard et al 2014)	Farmed pea, lentil and wheat in temperate semiarid prairie	454 sequencing of AMF SSU	Root is abundant in *Rhizopahgus* and *Funneliformis*, soil is abundant in *Paraglomus*
(Wilde et al 2009)	Two salt marshes in Germany	Both morphological and molecular criteria	*Rhizophagus* (*Glomus*) *intraradices* is more abundant in root,<br>*Funneliformis* (*Glomus*) *geosporum* is more abundant in soil
(Yang et al 2013)	Alpine meadow subjected to warming and grazing in China	Sanger sequencing of SSU-ITS-LSU	One Diversisporales was more abundant in soil, two Glomerales were more abundant in root
(Beauregard et al 2013)	Crop fields in Canada	DGGE	Six out of seven ribotypes show difference between root and soil
(Liu et al 2012)	Alpine Meadow subjected to fertilization in China	Soil spore isolation; Root, sanger sequencing of SSU	*Rhizophagus* was the most abundant AMF in root; *Diverspora* was the most abundant AMF in soil spores
(Hart and Reader 2002)	Pot experiment to test the colonization potential of 21 AMF isolates from three families	Percent of colonization and fungal biomass in root; Hyphal length in soil	Glomaceae isolates had high root colonization but low soil colonization, Gigasporaceae isolates showed the opposite trend whereas Acaulosporaceae isolates had low root and soil colonization.
(Verbruggen et al 2012)	40 agricultural soils in the Netherlands	tRFLP	No obvious different between root and soil AMF communities

(Wu et al 2007)	Primary successional volcanic desert in Japan	tRFLP	AMF community structures detected by spore sampling were inconsistent with those from plant roots.

SSU: small subunit; LSU, large subunit; ITS: internal transcribed spacer; tRFLP, terminal restriction fragment length polymorphism; DGGE: Denaturing Gradient Gel Electrophoresis.

Reference

Bahram M, Peay KG, Tedersoo L (2015). Local-scale biogeography and spatiotemporal variability in communities of mycorrhizal fungi. *New Phytol* **205:** 1454-1463.

Bainard LD, Koch AM, Gordon AM, Klironomos JN (2012). Temporal and compositional differences of arbuscular mycorrhizal fungal communities in conventional monocropping and tree-based intercropping systems. *Soil Biol Biochem* **45:** 172-180.

Bainard LD, Bainard JD, Hamel C, Gan Y (2014). Spatial and temporal structuring of arbuscular mycorrhizal communities is differentially influenced by abiotic factors and host crop in a semi-arid prairie agroecosystem. *FEMS Microbiol Ecol* **88:** 333-344.

Barnes CJ, Burns CA, van der Gast CJ, McNamara NP, Bending GD (2016a). Spatio-Temporal Variation of Core and Satellite Arbuscular Mycorrhizal Fungus Communities in Miscanthus giganteus. *Front Microbiol* **7:** 1278.

Barnes CJ, van der Gast CJ, Burns CA, McNamara NP, Bending GD (2016b). Temporally Variable Geographical Distance Effects Contribute to the Assembly of Root-Associated Fungal Communities. *Front Microbiol* **7:** 195.

Beauregard MS, Gauthier MP, Hamel C, Zhang T, Welacky T, Tan CS *et al* (2013). Various forms of organic and inorganic P fertilizers did not negatively affect soil- and root-inhabiting AM fungi in a maize–soybean rotation system. *Mycorrhiza* **23:** 143-154.

Bencherif K, Boutekrabt A, Dalpé Y, Lounès-Hadj Sahraoui A (2016). Soil and seasons affect arbuscular mycorrhizal fungi associated with Tamarix rhizosphere in arid and semi-arid steppes. *Appl Soil Ecol* **107:** 182-190.

Bennett AE, Daniell TJ, Öpik M, Davison J, Moora M, Zobel M *et al* (2013). Arbuscular Mycorrhizal Fungal Networks Vary throughout the Growing Season and between Successional Stages. *PLOS ONE* **8:** e83241.

Błaszkowski J, Renker C, Buscot F (2006). Glomus drummondii and G. walkeri, two new species of arbuscular mycorrhizal fungi (Glomeromycota). *Mycological Research* **110:** 555-566.

Bouffaud M-L, Bragalini C, Berruti A, Peyret-Guzzon M, Voyron S, Stockinger H *et al* (2017). Arbuscular mycorrhizal fungal community differences among European long-term observatories. *Mycorrhiza* **27:** 331-343.

Camenzind T, Hempel S, Homeier J, Horn S, Velescu A, Wilcke W *et al* (2014). Nitrogen and phosphorus additions impact arbuscular mycorrhizal abundance and molecular diversity in a tropical montane forest. *Glob Change Biol* **20:** 3646-3659.

Chagnon PL, Bradley RL, Klironomos JN (2012). Using ecological network theory to evaluate the causes and consequences of arbuscular mycorrhizal community structure. *New Phytol* **194:** 307-312.

Chen L, Zheng Y, Gao C, Mi X-C, Ma K-P, Wubet T *et al* (2017a). Phylogenetic relatedness explains highly interconnected and nested symbiotic networks of woody plants and arbuscular mycorrhizal fungi in a Chinese subtropical forest. *Mol Ecol* **26:** 2563-2575.

Chen YL, Xu ZW, Xu TL, Veresoglou SD, Yang GW, Chen BD (2017b). Nitrogen deposition and precipitation induced phylogenetic clustering of arbuscular mycorrhizal fungal communities. *Soil Biol Biochem* **115:** 233-242.

Daniell TJ, Husband R, Fitter AH, Young JPW (2001). Molecular diversity of arbuscular mycorrhizal fungi colonising arable crops. *FEMS Microbiol Ecol* **36:** 203-209.

Davison J, Öpik M, Zobel M, Vasar M, Metsis M, Moora M (2012). Communities of Arbuscular Mycorrhizal Fungi Detected in Forest Soil Are Spatially Heterogeneous but Do Not Vary throughout the Growing Season. *PLOS ONE* **7:** e41938.

Davison J, Moora M, Jairus T, Vasar M, Öpik M, Zobel M (2016). Hierarchical assembly rules in arbuscular mycorrhizal (AM) fungal communities. *Soil Biol Biochem* **97:** 63-70.

Dumbrell AJ, Ashton PD, Aziz N, Feng G, Nelson M, Dytham C *et al* (2011). Distinct seasonal assemblages of arbuscular mycorrhizal fungi revealed by massively parallel pyrosequencing. *New Phytol* **190:** 794-804.

Egan CP, Callaway RM, Hart MM, Pither J, Klironomos J (2017). Phylogenetic structure of arbuscular mycorrhizal fungal communities along an elevation gradient. *Mycorrhiza* **27:** 273-282.

Fadrosh DW, Ma B, Gajer P, Sengamalay N, Ott S, Brotman RM *et al* (2014). An improved dual-indexing approach for multiplexed 16S rRNA gene sequencing on the Illumina MiSeq platform. *Microbiome* **2:** 6.

García de León D, Moora M, Öpik M, Jairus T, Neuenkamp L, Vasar M *et al* (2016a). Dispersal of arbuscular mycorrhizal fungi and plants during succession. *Acta Oecol-Int J Ecol* **77:** 128-135.

García de León D, Moora M, Öpik M, Neuenkamp L, Gerz M, Jairus T *et al* (2016b). Symbiont dynamics during ecosystem succession: co-occurring plant and arbuscular mycorrhizal fungal communities. *FEMS Microbiol Ecol* **92**.

Gorzelak MA, Pickles BJ, Hart MM (2017). Exploring the symbiont diversity of ancient western redcedars: arbuscular mycorrhizal fungi of long-lived hosts. *Mol Ecol* **26:** 1586-1597.

Guadarrama P, Castillo S, Ramos-Zapata JA, Hernández-Cuevas LV, Camargo-Ricalde SL (2014). Arbuscular mycorrhizal fungal communities in changing environments: The effects of seasonality and anthropogenic disturbance in a seasonal dry forest. *Pedobiologia* **57:** 87-95.

Hart MM, Reader RJ (2002). Taxonomic basis for variation in the colonization strategy of arbuscular mycorrhizal fungi. *New Phytol* **153:** 335-344.

Hart MM, Gorzelak M, Ragone D, Murch SJ (2014). Arbuscular mycorrhizal fungal succession in a long-lived perennial. *Botany* **92:** 313-320.

Hazard C, Boots B, Keith AM, Mitchell DT, Schmidt O, Doohan FM *et al* (2014). Temporal variation outweighs effects of biosolids applications in shaping arbuscular mycorrhizal fungi communities on plants grown in pasture and arable soils. *Appl Soil Ecol* **82:** 52-60.

Helgason T, Fitter AH, Young JPW (1999). Molecular diversity of arbuscular mycorrhizal fungi colonising Hyacinthoides non-scripta (bluebell) in a seminatural woodland. *Mol Ecol* **8:** 659-666.

Helgason T, Feng H, Sherlock DJ, Young JPW, Fitter AH (2014). Arbuscular mycorrhizal communities associated with maples (Acer spp.) in a common garden are influenced by season and host plant. *Botany* **92:** 321-326.

Hempel S, Renker C, Buscot F (2007). Differences in the species composition of arbuscular mycorrhizal fungi in spore, root and soil communities in a grassland ecosystem. *Environ Microbiol* **9:** 1930-1938.

Herrmann L, Lesueur D, Bräu L, Davison J, Jairus T, Robain H *et al* (2016). Diversity of root-associated arbuscular mycorrhizal fungal communities in a rubber tree plantation chronosequence in Northeast Thailand. *Mycorrhiza* **26:** 863-877.

Higo M, Isobe K, Drijber RA, Kondo T, Yamaguchi M, Takeyama S *et al* (2014). Impact of a 5-year winter cover crop rotational system on the molecular diversity of arbuscular mycorrhizal fungi colonizing roots of subsequent soybean. *Biol Fertil Soils* **50:** 913-926.

Higo M, Isobe K, Kondo T, Yamaguchi M, Takeyama S, Drijber RA *et al* (2015). Temporal variation of the molecular diversity of arbuscular mycorrhizal communities in three different winter cover crop rotational systems. *Biol Fertil Soils* **51:** 21-32.

Honnay O, Helsen K, Van Geel M (2017). Plant community reassembly on restored semi-natural grasslands lags behind the assembly of the arbuscular mycorrhizal fungal communities. *Biol Conserv* **212:** 196-208.

Horn S, Caruso T, Verbruggen E, Rillig MC, Hempel S (2014). Arbuscular mycorrhizal fungal communities are phylogenetically clustered at small scales. *ISME J* **8:** 2231-2242.

Husband R, Herre EA, Turner SL, Gallery R, Young JPW (2002a). Molecular diversity of arbuscular mycorrhizal fungi and patterns of host association over time and space in a tropical forest. *Mol Ecol* **11:** 2669-2678.

Husband R, Herre EA, Young JPW (2002b). Temporal variation in the arbuscular mycorrhizal communities colonising seedlings in a tropical forest. *FEMS Microbiol Ecol* **42:** 131-136.

Johnson NC, Zak DR, Tilman D, Pfleger F (1991). Dynamics of vesicular-arbuscular mycorrhizae during old field succession. *Oecologia* **86:** 349-358.

Kabir Z, O'Halloran IP, Fyles JW, Hamel C (1997). Seasonal changes of arbuscular mycorrhizal fungi as affected by tillage practices and fertilization : Hyphal density and mycorrhizal root colonization. *Plant Soil* **192:** 285-293.

Kawahara A, An GH, Miyakawa S, Sonoda J, Ezawa T (2016). Nestedness in Arbuscular Mycorrhizal Fungal Communities along Soil pH Gradients in Early Primary Succession: Acid-Tolerant Fungi Are pH Generalists. *PLOS ONE* **11:** e0165035.

Kivlin SN, Hawkes CV, Treseder KK (2011). Global diversity and distribution of arbuscular mycorrhizal fungi. *Soil Biol Biochem* **43:** 2294-2303.

Kivlin SN, Hawkes CV (2016). Tree species, spatial heterogeneity, and seasonality drive soil fungal abundance, richness, and composition in Neotropical rainforests. *Environ Microbiol* **18:** 4662-4673.

Kowalchuk GA, De Souza FA, Van Veen JA (2002). Community analysis of arbuscular mycorrhizal fungi associated with Ammophila arenaria in Dutch coastal sand dunes. *Mol Ecol* **11:** 571-581.

Koziol L, Bever JD (2016). AMF, phylogeny, and succession: specificity of response to mycorrhizal fungi increases for late-successional plants. *Ecosphere* **7**.

Krüger C, Kohout P, Janoušková M, Püschel D, Frouz J, Rydlová J (2017). Plant communities rather than soil properties structure arbuscular mycorrhizal fungal communities along primary succession on a mine spoil. *Front Microbiol* **8**.

Krüger M, Krüger C, Walker C, Stockinger H, Schüßler A (2012). Phylogenetic reference data for systematics and phylotaxonomy of arbuscular mycorrhizal fungi from phylum to species level. *New Phytol* **193:** 970-984.

Krüger M, Teste FP, Laliberté E, Lambers H, Coghlan M, Zemunik G *et al* (2015). The rise and fall of arbuscular mycorrhizal fungal diversity during ecosystem retrogression. *Mol Ecol* **24:** 4912-4930.

Liu W, Zhang YL, Jiang SS, Deng Y, Christie P, Murray PJ *et al* (2016). Arbuscular mycorrhizal fungi in soil and roots respond differently to phosphorus inputs in an intensively managed calcareous agricultural soil. *Sci Rep* **6:** 11.

Liu Y, He L, An L, Helgason T, Feng H (2009). Arbuscular mycorrhizal dynamics in a chronosequence of Caragana korshinskii plantations. *FEMS Microbiol Ecol* **67:** 81-92.

Liu Y, Shi G, Mao L, Cheng G, Jiang S, Ma X *et al* (2012). Direct and indirect influences of 8 yr of nitrogen and phosphorus fertilization on Glomeromycota in an alpine meadow ecosystem. *New Phytol* **194:** 523-535.

Liu Y, Johnson NC, Mao L, Shi G, Jiang S, Ma X *et al* (2015a). Phylogenetic structure of arbuscular mycorrhizal community shifts in response to increasing soil fertility. *Soil Biol Biochem* **89:** 196-205.

Liu Y, Mao L, Li J, Shi G, Jiang S, Ma X *et al* (2015b). Resource availability differentially drives community assemblages of plants and their root-associated arbuscular mycorrhizal fungi. *Plant Soil* **386:** 341-355.

López-García Á, Palenzuela J, Barea JM, Azcón-Aguilar C (2014). Life-history strategies of arbuscular mycorrhizal fungi determine succession into roots of Rosmarinus officinalis L., a characteristic woody perennial plant species from Mediterranean ecosystems. *Plant Soil* **379:** 247-260.

Maherali H, Klironomos JN (2007). Influence of Phylogeny on Fungal Community Assembly and Ecosystem Functioning. *Science* **316:** 1746-1748.

Maherali H, Klironomos JN (2012). Phylogenetic and Trait-Based Assembly of Arbuscular Mycorrhizal Fungal Communities. *PLOS ONE* **7:** e36695.

Martínez-García LB, Richardson SJ, Tylianakis JM, Peltzer DA, Dickie IA (2015). Host identity is a dominant driver of mycorrhizal fungal community composition during ecosystem development. *New Phytol* **205:** 1565-1576.

Montero Sommerfeld H, Díaz LM, Alvarez M, Añazco Villanueva C, Matus F, Boon N *et al* (2013). High winter diversity of arbuscular mycorrhizal fungal communities in shallow and deep grassland soils. *Soil Biol Biochem* **65:** 236-244.

Montesinos-Navarro A, Segarra-Moragues JG, Valiente-Banuet A, Verdú M (2012). The network structure of plant–arbuscular mycorrhizal fungi. *New Phytol* **194:** 536-547.

Mueller RC, Bohannan BJM (2015). Shifts in the phylogenetic structure of arbuscular mycorrhizal fungi in response to experimental nitrogen and carbon dioxide additions. *Oecologia* **179:** 175-185.

Oba H, Shinozaki N, Oyaizu H, Tawaraya K, Wagatsuma T, Barraquio WL *et al* (2004). Arbuscular mycorrhizal fungal communities associated with some pioneer plants in the lahar area of Mt. Pinatubo, Philippines. *Soil Sci Plant Nutr* **50:** 1195-1203.

Oehl F, Schneider D, Sieverding E, Burga CA (2011). Succession of arbuscular mycorrhizal communities in the foreland of the retreating Morteratsch glacier in the Central Alps. *Pedobiologia* **54:** 321-331.

Pezzani F, Montaña C, Guevara R (2006). Associations between arbuscular mycorrhizal fungi and grasses in the successional context of a two-phase mosaic in the Chihuahuan Desert. *Mycorrhiza* **16:** 285-295.

Roger A, Colard A, Angelard C, Sanders IR (2013). Relatedness among arbuscular mycorrhizal fungi drives plant growth and intraspecific fungal coexistence. *ISME J* **7:** 2137-2146.

Roy J, Reichel R, Brüggemann N, Hempel S, Rillig MC (2017). Succession of arbuscular mycorrhizal fungi along a 52-year agricultural recultivation chronosequence. *FEMS Microbiol Ecol* **93:** fix102.

Saks Ü, Davison J, Öpik M, Vasar M, Moora M, Zobel M (2013). Root-colonizing and soil-borne communities of arbuscular mycorrhizal fungi in a temperate forest understorey. *Botany* **92:** 277-285.

Senés-Guerrero C, Schüßler A (2016). A conserved arbuscular mycorrhizal fungal core-species community colonizes potato roots in the Andes. *Fungal Diversity* **77:** 317-333.

Sheng M, Chen X, Zhang X, Hamel C, Cui X, Chen J *et al* (2017). Changes in arbuscular mycorrhizal fungal attributes along a chronosequence of black locust (Robinia pseudoacacia)

plantations can be attributed to the plantation-induced variation in soil properties. *Sci Total Environ* **599:** 273-283.

Shi G, Yao B, Liu Y, Jiang S, Wang W, Pan J *et al* (2017). The phylogenetic structure of AMF communities shifts in response to gradient warming with and without winter grazing on the Qinghai–Tibet Plateau. *Appl Soil Ecol* **121:** 31-40.

Sikes BA, Maherali H, Klironomos JN (2012). Arbuscular mycorrhizal fungal communities change among three stages of primary sand dune succession but do not alter plant growth. *Oikos* **121:** 1791-1800.

Taylor DL, Hollingsworth TN, McFarland JW, Lennon NJ, Nusbaum C, Ruess RW (2014). A first comprehensive census of fungi in soil reveals both hyperdiversity and fine-scale niche partitioning. *Ecol Monogr* **84:** 3-20.

Taylor DL, Walters WA, Lennon NJ, Bochicchio J, Krohn A, Caporaso JG *et al* (2016). Accurate estimation of fungal diversity and abundance through improved lineage-specific primers optimized for Illumina amplicon sequencing. *Appl Environ Microbiol* **82:** 7217-7226.

Toju H, Guimarães PR, Olesen JM, Thompson JN (2014). Assembly of complex plant–fungus networks. *Nat Commun* **5**.

Turrini A, Sbrana C, Avio L, Njeru EM, Bocci G, Bàrberi P *et al* (2016). Changes in the composition of native root arbuscular mycorrhizal fungal communities during a short-term cover crop-maize succession. *Biol Fertil Soils* **52:** 643-653.

Vályi K, Rillig MC, Hempel S (2015). Land-use intensity and host plant identity interactively shape communities of arbuscular mycorrhizal fungi in roots of grassland plants. *New Phytol* **205:** 1577-1586.

van Geel M, Ceustermans A, van Hemelrijck W, Lievens B, Honnay O (2015). Decrease in diversity and changes in community composition of arbuscular mycorrhizal fungi in roots of apple trees with increasing orchard management intensity across a regional scale. *Mol Ecol* **24:** 941-952.

Van Geel M, Jacquemyn H, Plue J, Saar L, Kasari L, Peeters G *et al* (2017a). Abiotic rather than biotic filtering shapes the arbuscular mycorrhizal fungal communities of European seminatural grasslands. *New Phytologist*: n/a-n/a.

Van Geel M, Verbruggen E, De Beenhouwer M, van Rennes G, Lievens B, Honnay O (2017b). High soil phosphorus levels overrule the potential benefits of organic farming on arbuscular mycorrhizal diversity in northern vineyards. *Agriculture, Ecosystems & Environment* **248:** 144-152.

Varela-Cervero S, López-García Á, Barea JM, Azcón-Aguilar C (2016). Spring to autumn changes in the arbuscular mycorrhizal fungal community composition in the different propagule types associated to a Mediterranean shrubland. *Plant Soil* **408:** 107-120.

Verbruggen E, Van Der Heijden MGA, Weedon JT, Kowalchuk GA, RÖLing WFM (2012). Community assembly, species richness and nestedness of arbuscular mycorrhizal fungi in agricultural soils. *Mol Ecol* **21:** 2341-2353.

Voříšková J, Brabcová V, Cajthaml T, Baldrian P (2014). Seasonal dynamics of fungal communities in a temperate oak forest soil. *New Phytol* **201:** 269-278.

Wilde P, Manal A, Stodden M, Sieverding E, Hildebrandt U, Bothe H (2009). Biodiversity of arbuscular mycorrhizal fungi in roots and soils of two salt marshes. *Environ Microbiol* **11:** 1548-1561.

Wu B, Hogetsu T, Isobe K, Ishii R (2007). Community structure of arbuscular mycorrhizal fungi in a primary successional volcanic desert on the southeast slope of Mount Fuji. *Mycorrhiza* **17:** 495-506.

Yang W, Zheng Y, Gao C, He X, Ding Q, Kim Y *et al* (2013). The Arbuscular Mycorrhizal Fungal Community Response to Warming and Grazing Differs between Soil and Roots on the Qinghai-Tibetan Plateau. *PLOS ONE* **8:** e76447.

Yu J, Xue Z, He X, Liu C, Steinberger Y (2017). Shifts in composition and diversity of arbuscular mycorrhizal fungi and glomalin contents during revegetation of desertified semiarid grassland. *Appl Soil Ecol* **115:** 60-67.

Yu L, Nicolaisen M, Larsen J, Ravnskov S (2012). Succession of root-associated fungi in Pisum sativum during a plant growth cycle as examined by 454 pyrosequencing. *Plant Soil* **358:** 225-233.

Zeng H, Tan F, Zhang Y, Feng Y, Shu Y, Wang J (2014). Effects of cultivation and return of Bacillus thuringiensis (Bt) maize on the diversity of the arbuscular mycorrhizal community in soils and roots of subsequently cultivated conventional maize. *Soil Biol Biochem* **75:** 254-263.
